# Extensive bidirectional genetic overlap between bipolar disorder and cardiovascular disease phenotypes

**DOI:** 10.1038/s41398-021-01527-z

**Published:** 2021-07-23

**Authors:** Linn Rødevand, Shahram Bahrami, Oleksandr Frei, Yunhan Chu, Alexey Shadrin, Kevin S. O’Connell, Olav B. Smeland, Torbjørn Elvsåshagen, Guy F. L. Hindley, Srdjan Djurovic, Anders M. Dale, Trine V. Lagerberg, Nils Eiel Steen, Ole A. Andreassen

**Affiliations:** 1 NORMENT, Centre for Mental Disorders Research, Division of Mental Health and Addiction, Oslo University Hospital, and Institute of Clinical Medicine, University of Oslo, Oslo, Norway; 2 Center for Bioinformatics, Department of Informatics, University of Oslo, Oslo, Norway; 3 Department of Neurology, Oslo University Hospital, Oslo, Norway; 4 Institute of Psychiatry, Psychology and Neuroscience, King’s College London, London, UK; 5 Department of Medical Genetics, Oslo University Hospital, Oslo, Norway; 6 NORMENT Centre, Department of Clinical Science, University of Bergen, Bergen, Norway; 7 Department of Radiology, University of California San Diego, La Jolla, CA USA; 8 Multimodal Imaging Laboratory, University of California San Diego, La Jolla, CA USA; 9 Department of Psychiatry, University of California San Diego, La Jolla, CA USA; 10 Department of Neurosciences, University of California San Diego, La Jolla, CA USA

**Keywords:** Personalized medicine, Predictive markers, Psychiatric disorders

## Abstract

Patients with bipolar disorder (BIP) have a high risk of cardiovascular disease (CVD), despite considerable individual variation. The mechanisms underlying comorbid CVD in BIP remain largely unknown. We investigated polygenic overlap between BIP and CVD phenotypes, including CVD risk factors and coronary artery disease (CAD). We analyzed large genome-wide association studies of BIP (*n* = 51,710) and CVD phenotypes (*n* = 159,208–795,640), using bivariate causal mixture model (MiXeR), which estimates the total amount of shared genetic variants, and conjunctional false discovery rate (FDR), which identifies specific overlapping loci. MiXeR revealed polygenic overlap between BIP and body mass index (BMI) (82%), diastolic and systolic blood pressure (20–22%) and CAD (11%) despite insignificant genetic correlations. Using conjunctional FDR < 0.05, we identified 129 shared loci between BIP and CVD phenotypes, mainly BMI (*n* = 69), systolic (*n* = 53), and diastolic (*n* = 53) blood pressure, of which 22 are novel BIP loci. There was a pattern of mixed effect directions of the shared loci between BIP and CVD phenotypes. Functional analyses indicated that the shared loci are linked to brain-expressed genes and involved in neurodevelopment, lipid metabolism, chromatin assembly/disassembly and intracellular processes. Altogether, the study revealed extensive polygenic overlap between BIP and comorbid CVD, implicating shared molecular genetic mechanisms. The mixed effect directions of the shared loci suggest variation in genetic susceptibility to CVD across BIP subgroups, which may underlie the heterogeneity of CVD comorbidity in BIP patients. The findings suggest more focus on targeted lifestyle interventions and personalized pharmacological treatment to reduce CVD comorbidity in BIP.

## Introduction

People with bipolar disorder (BIP) have on average twice as high risk of cardiovascular disease (CVD) compared to the general population, contributing to a reduction in life expectancy [1–3]. CVD comorbidity and mortality have remained high during the past decades, indicating that most patients with BIP have not benefited from recent advances in medicine [4–7]. The etiology of the CVD comorbidity remains largely unknown, but it is likely to be associated with medication side-effects and lifestyle factors, such as poor diet, physical inactivity, and smoking [1, 8]. A genetic susceptibility to CVD may also play a role, similar to what has been indicated in schizophrenia [9, 10], including overlapping genetic loci [11, 12]. This is supported by the considerable genetic overlap between schizophrenia and BIP [13]. However, there is a large individual variation in CVD comorbidity [2–4], which suggests increased genetic risk for CVD in subgroups of BIP.

BIP is a complex disorder with heritability estimates of 70–80% [14]. The polygenic nature of BIP is becoming increasingly apparent as recent genome-wide association studies (GWASs) have identified 64 risk loci for BIP [15]. GWASs have also discovered many genetic loci associated with CVD risk factors, including body mass index (BMI) [16, 17], type 2 diabetes (T2D) [18], total cholesterol (TC) [19], low-density lipoprotein (LDL) cholesterol [19], high-density lipoprotein (HDL) cholesterol [19], systolic blood pressure (SBP) [20], diastolic blood pressure (DBP) [20], along with coronary artery disease (CAD) [21].

Few studies have investigated the genetic relationship between BIP and CVD risk factors and CAD [22–24]. A recent study suggested an inverse genetic relationship between BIP and CVD risk factors (BMI, TC, LDL, HDL) [23], indicating that BIP may be related to reduced genetic risk of CVD. However, the results varied depending on using polygenic risk scores (PRS) or linkage disequilibrium score regression (LDSR) [23]; the latter did not provide significant results. Importantly, a significant genetic correlation estimated with LDSR requires consistent effect directions of the shared variants between the phenotypes [25]. Thus, genetic correlation fails to capture polygenic overlap in the presence of a mixture of effect directions across shared variants [26]. The bivariate causal mixture model (MiXeR), which estimates the total number of shared genetic variants [27], can identify polygenic overlap (i.e., shared genetic architecture among common variants), beyond genetic correlations. Further, the conditional/conjunctional false discovery rate (cond/conjFDR) methodology can identify the specific overlapping loci [28]. These methods have the advantage of identifying shared variants regardless of their effect directions [27, 28]. In addition, the cond/conjFDR tools increase the power for genetic discovery due to joint analysis of two GWAS, leading to the identification of loci that do not reach significance threshold in traditional GWAS analyses [28], as illustrated with several complex human traits [12, 24, 29].

We recently discovered 69 shared loci between BIP and BMI, of which 52% possessed concordant effect directions, while genetic correlation was insignificant [24]. These results demonstrate polygenic overlap between BIP and BMI and the mixed effect directions may suggest subgroups of BIP with higher susceptibility for weight gain. Further, the findings highlight the importance of analysis of genetic correlation with analytical methods that allow for identification of shared genetic variants irrespective of their effect directions [26].

In the present study, we investigated the polygenic overlap between BIP, CVD risk factors, and CAD beyond genetic correlations with the MiXeR method [27], and applied the cond/conjFDR approach to identify specific shared loci [28]. We expect to unravel more of the shared genetic architecture between BIP, CVD risk factors, and CAD, and enhance the discovery of specific overlapping genetic loci to inform the underlying molecular mechanisms.

## Methods

### Participant samples

We obtained GWAS results in the form of summary statistics (*p*-values and *z*-scores). BIP data were retrieved from Psychiatric Genomics Consortium (PGC) and consisted of 20,352 cases and 31,358 controls from 32 samples [30]. Among the cases, 14,879 individuals were diagnosed with BIP type I, 3421 with BIP type II, 977 with schizoaffective disorder, bipolar type), and the remaining BIP not otherwise specified [30]. Further, we used data from large GWASs on CVD phenotypes, including BMI, TC, HDL, LDL, SBP, DBP, and T2D and CAD (*n* = 159,208–795,640) [17–21]. We repeated the previously published analysis of genetic overlap between BIP and BMI [24] using cond/conjFDR. While MiXeR corrects for overlapping samples [27], cond/conjFDR does not [28]. Thus, we screened for overlapping samples between the BIP GWAS and the CVD GWASs by checking the substudies included in the GWASs, and found no overlapping samples. However, we did not have access to individual genotype data and were thus prevented from determining whether any individuals participated in both the BIP GWAS and any of the CVD GWASs. For further information about the GWASs, see Supplementary Methods and original publications [17–21, 30]. The local ethics committees approved all GWASs used in the current study, and all participants provided informed consent. Regional Committees for Medical Research Ethics - South-East Norway has evaluated the current protocol and found that no additional institutional review board approval was necessary because no individual data were used.

### Statistical analysis

We constructed conditional quantile–quantile (Q–Q) plots to visualize the putative overlap in SNPs associations, i.e., cross-trait enrichment. Enrichment exists when the proportion of SNPs associated with a phenotype (e.g., BIP) increases as a function of the strength of the association with a secondary phenotype (e.g., BMI) [28]. In the conditional Q–Q plots, this cross-trait enrichment is visualized as successive leftward shifts from the null line [12, 28]. Details about this method are available in Supplementary Methods.

Next, we used the statistical tool MiXeR to estimate the total number of shared and unique trait-influencing variants (i.e., variants with pure genetic effects not induced by LD) using GWAS summary data [27]. This method evaluates polygenic overlap independent of genetic correlation between phenotypes. The MiXeR results are illustrated with Venn diagrams of shared and unique variants. Estimates of uncertainty are provided, including standard error in parenthesis in the Venn diagrams. We evaluated the model fit, i.e., the ability of the MiXeR model to predict the actual GWAS data, based on modelled vs. actual conditional Q–Q plots, negative log-likelihood plots, and Akaike information criterion (AIC). The more closely the model-based Q–Q plots follow the actual Q–Q plots, the better the MiXeR predicts the data, indicating more precise estimates. The negative log-likelihood plots visualize the performance of the best model versus models with minimum and maximum polygenic overlap. More specifically, the best model with polygenic overlap estimated with MiXeR was compared with two models—a model with least possible overlap and a model with maximum possible overlap. The lowest point on the negative log-likelihood curve indicates better model fit. Support for the MiXeR model is a clearly defined minimum on the negative log-likelihood curve, as quantified by AIC criteria. A positive AIC value yields support for the MiXeR model of polygenic overlap and suggests that the GWAS data has enough power to distinguish the estimated polygenic overlap using MiXeR from the constrained models with minimal and maximum polygenic overlap [27]. For details about MiXeR, see Supplementary Methods and Frei et al. [27].

The condFDR approach was used to increase discovery of specific genetic variants associated with BIP and CVD phenotypes [28]. The condFDR method builds on Bayesian statistics and increases the power to identify loci associated with a primary phenotype (e.g., BIP) by leveraging associations with a secondary phenotype (e.g., BMI). Thus, this method re-ranks the test-statistics of a primary phenotype (e.g., BIP) based on a conditional variable, i.e., the strength of the association with a secondary phenotype (e.g., BMI) [28]. Inverting the roles of primary and secondary phenotypes yields the inverse condFDR value [28]. ConjFDR is an extension of condFDR and can detect loci jointly associated with two phenotypes (e.g., both BIP and BMI) [28]. ConjFDR is defined as the maximum of the two condFDR values, providing a conservative estimate of the FDR for a SNP association with both phenotypes [12, 28]. *P*-values are corrected for inflation using a genomic inflation control procedure [12, 28]. Consistent with previous publications [12, 24, 31, 32], we used the thresholds condFDR<0.01 and conjFDR<0.05. For further information, see Supplementary Methods and method review [28].

### Genomic loci definition

To define the independent genomic loci, we applied FUMA, an online tool for functional mapping of genetic variants (http://fuma.ctglab.nl/) [33]. Independent significant SNPs were defined as SNPs with condFDR<0.01 or conjFDR<0.05 and independent from each other at LD *r*^2^ < 0.6. Lead SNPs were identified by retaining those independent significant SNPs that were independent from each other at *r*^*2*^ < 0.1. To define distinct genomic loci, we merged any physically overlapping lead SNPs (LD blocks <250 kb apart) selecting a SNP with the lowest *p*-value as a lead SNP of the merged locus. The borders of the genomic loci were defined by identifying all SNPs (candidate SNPs) in LD (*r*^2^ ≧ 0.6) with one of the independent significant SNPs in the locus [33] (see Supplementary Methods).

### Effect directions and genetic correlations

We evaluated the directional effects of the shared lead SNPs between BIP and CVD phenotypes by comparing their *z-*scores and odds ratios from the original publications [16, 18–21, 30]. Genetic correlations were estimated using LDSR and corrected for multiple testing (0.05/8) [34].

### Functional annotation

We used FUMA [33] to functionally annotate candidate SNPs in the genomic loci with a condFDR/conjFDR value <0.10 and an LD *r*^2^ ≧ 0.6 with one of the independent significant SNPs. SNPs were annotated using three different tools, including Combined Annotation Dependent Depletion (CADD) [35], a method that predicts the deleteriousness of SNPs on protein structure/function; RegulomeDB [36], which predicts regulatory functions; and chromatin states that indicate the transcription/regulation effects at the SNP locus [37, 38]. We also identified previously reported GWAS associations in the GWAS catalog [39] overlapping with the identified loci. We proceeded with further functional analyses provided that we identified at least one shared locus at conjFDR<0.05. Thus, the prerequisite for performing functional analysis was the presence of ≥ one locus jointly associated with BIP and a given CVD phenotype. The functional analyses included gene-mapping, gene-set enrichment analysis, pathway analysis, and spatio-temporal analysis of gene expression. In particular, FUMA was used to map lead and candidate SNPs to genes based on either of three properties of the SNPs: (1) their physical position (i.e., proximity to a gene), (2) expression quantitative trait locus (eQTL) functionality, and (3) chromatin interaction [33]. Next, we investigated whether genes mapped to all SNPs in shared loci were overrepresented in gene-sets using FUMA [33] and in pathways using ConsensusPathDB [40]. We also performed gene-set analysis of genes nearest to the lead SNPs in the shared loci at conjFDR<0.05. Finally, we run spatio-temporal analysis of genes mapped to SNPs in the shared loci between BIP and CVD phenotypes to investigate patterns of expression across brain tissues and through developmental periods [41–44]. For details, see Supplementary Methods.

## Results

### Genetic overlap between BIP and CVD phenotypes

In the conditional Q–Q plots, we observed enrichment in BIP SNPs as a function of the significance of associations with CVD phenotypes (Supplementary Fig. 1), indicating polygenic overlap. The reverse conditional Q–Q plots also demonstrated enrichment in CVD phenotypes given associations with BIP (Supplementary Fig. 2).

After observing cross-trait enrichment, we applied MiXeR which discovered different polygenicity of BIP (8.1k), BMI (11k), SBP (4.4k), DBP (3.9k), and CAD (1.4k).

**Fig. 1:**
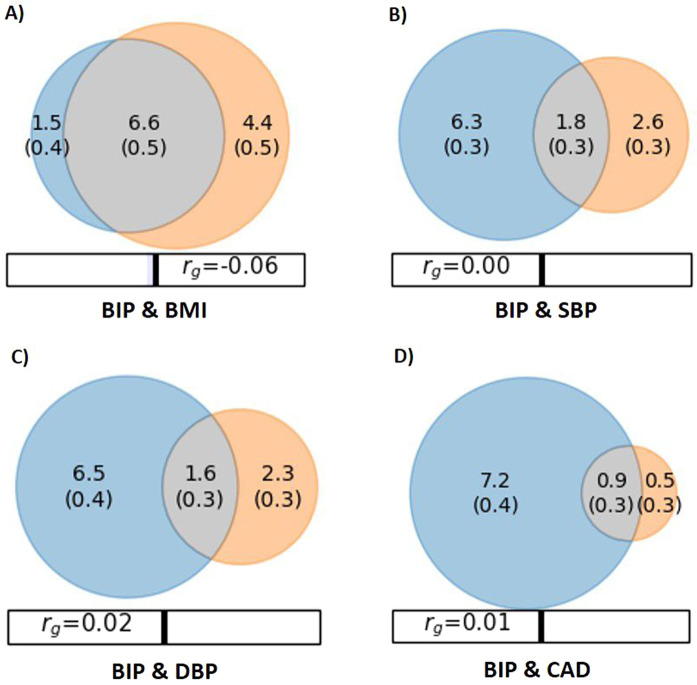
Venn diagrams of shared and unique polygenic variants. Venn diagrams of shared and unique trait-influencing variants, showing polygenic overlap (gray) between bipolar disorder (BIP) (blue) and (**A**) body mass index (BMI) (orange), (**B**) systolic blood pressure (SBP) (orange), (**C**) diastolic blood pressure (DBP) (orange), and (**D**) coronary artery disease (CAD) (orange). The numbers in the Venn diagram indicate the estimated quantity of shared and unique trait-influencing variants (in thousands), explaining 90% of SNP heritability in each phenotype, followed by standard error. The size of the circles reflects the degree of polygenicity. The figure is based on MiXeR results.

Parameter estimates of the MiXeR model and corresponding standard error are provided in Table 1 and Fig. 1. MiXeR revealed polygenic overlap between BIP and BMI, sharing 6.6k of 12.5k variants, as illustrated by the Venn diagram (Fig. 1A). The shared variants constitute 81.5 and 60% of variants influencing BIP and BMI, respectively. MiXeR also revealed polygenic overlap between BIP and SBP, sharing 1.8k of 10.7k variants (Fig. 1B), representing 22.2% and 40.9% of variants influencing BIP and SBP, respectively. Similarly, MiXeR identified polygenic overlap with DBP, sharing 1.6k of 10.4 K variants (Fig. 1C), constituting 19.8% and 41.0% of the genetic variants underlying BIP and DBP, respectively. In addition, BIP shared 0.9k of 8.6k variants with CAD (Fig. 1D), representing 11.1% and 64.3% of the genetic basis of BIP and CAD, respectively. Model fit was considered adequate as indicated by model-based Q–Q plots following the actual Q–Q plots (Supplementary Figures 3–6), although some caution in interpreting the MiXeR model for BIP and CAD is needed as the predicted Q–Q plots followed the observed Q–Q plots less closely at smaller *p*-values. The log-likelihood plots illustrated adequate model fit (Supplementary Figures 3–6) and AIC demonstrated sufficiently powered model (Supplementary MiXeR Table). The MiXeR model was not used for the other CVD phenotypes due to inadequate model fit (Supplementary Figures 7a–d).

**Table 1:** The results of MiXeR analysis for BIP and CVD phenotypes.

**Trait 1**	**Trait 2**	**Shared variants (s.e.)**	**Unique variants trait 1 (s.e.)**	**Unique variants trait 2 (s.e.)**
BIP	BMI	6.6 (0.5)	1.5 (0.4)	4.4 (0.5)
BIP	SBP	1.8 (0.3)	6.3 (0.3)	2.6 (0.3)
BIP	DBP	1.6 (0.3)	6.5 (0.4)	2.3 (0.3)
BIP	CAD	0.9 (0.3)	7.2 (0.4)	0.5 (0.3)

Number of shared and unique trait-influencing variants (in thousands), followed by standard error, estimated by MiXeR.

*BIP* bipolar disorder, *BMI* body mass index, *SBP* systolic blood pressure, *DBP* diastolic blood pressure, *CAD* coronary heart disease, *s.e.* standard error.

### Loci shared between BIP and CVD phenotypes

**Fig. 2:**
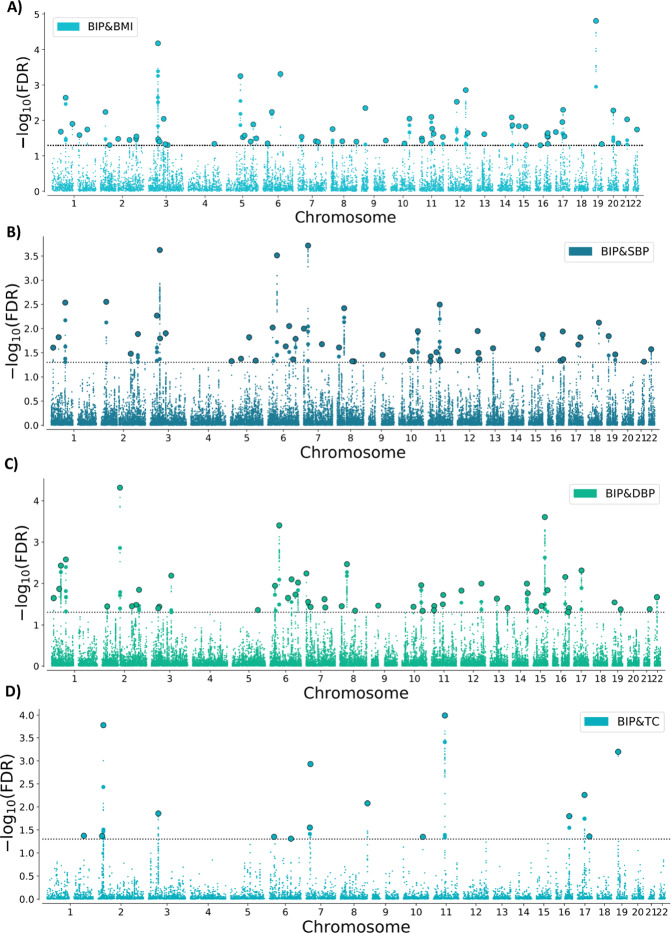

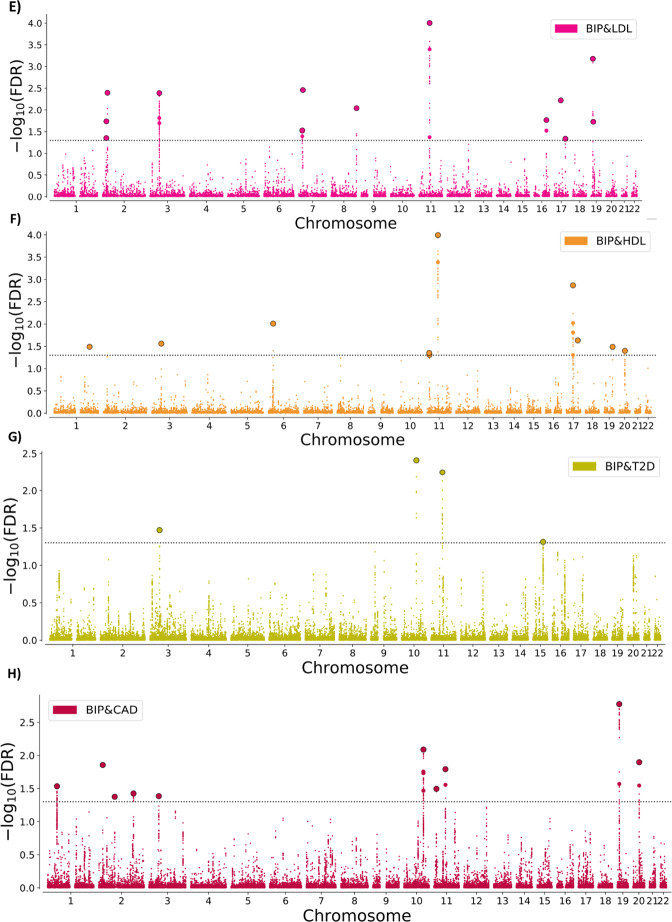
Common genetic variants jointly associated with bipolar disorder and cardiovascular disease phenotypes at conjFDR<0.05. Common genetic variants associated with both BIP and (**A**) BMI, (**B**) SBP, (**C**) DBP, (**D**) TC, (**E**) LDL, (**F**) HDL, (**G**) T2D, and (**H**) CAD at conjFDR<0.05. Manhattan plot showing the –log10 transformed conjFDR values for each SNP on the *y*-axis and chromosomal positions along the *x*-axis. SNPs with conjunction FDR<0.05 (i.e., −log10 FDR>1.3) are shown with enlarged data points. A black circle around the enlarged data points indicates the most significant SNP in each LD block. Further details are provided in Supplementary Tables. BIP bipolar disorder, CVD cardiovascular disease, BMI body mass index, SBP systolic blood pressure, DBP diastolic blood pressure, TC total cholesterol, LDL low-density lipoprotein cholesterol, HDL high-density lipoprotein cholesterol, T2D type 2 diabetes, CAD coronary artery disease, conjFDR conjunctional FDR. The results of BIP & BMI at conjFDR<0.01 are previously presented in Bahrami et al. [24].

At condFDR<0.01, we identified multiple loci associated with BIP conditional on their association with each CVD phenotype (Supplementary Tables 1–8), and vice versa (Supplementary Tables 9–16 and Supplementary Results). At conjFDR<0.05, we discovered several loci jointly associated with BIP and CVD phenotypes, including 69 loci shared with BMI as previously reported [24], and 53 loci with SBP, 53 loci with DBP, 15 with TC, 13 loci with LDL, 10 loci with HDL, 4 loci with T2D and 10 loci with CAD (Fig. 2A–H; Supplementary Tables 17–24). We observed small SNP *p*-values for both phenotypes, which indicate true associations with both BIP and CVD phenotypes. Several loci were jointly associated with BIP and more than one CVD phenotype, resulting in 129 distinct loci associated with both BIP and CVD phenotypes at conjFDR<0.05. Twenty-two of the shared loci are novel BIP loci (Supplementary Table 25). See Supplementary Methods for all the studies reviewed to determine the number of novel BIP loci.

We evaluated the directionality of allelic effects of the shared lead SNPs between the phenotypes by investigating their *z*-scores. There was a pattern of mixed effect directions of the shared SNPs between BIP and CVD risk factors (Table 2). We discovered the same effect direction of 52.2% of SNPs shared with BMI (as previously reported [24]), 49.1% SNPs shared with SBP, 47.2% SNPs shared with DBP, 26.7% SNPs shared with TC, 46.2% SNPs shared with LDL, 40% SNPs shared with HDL, 25% SNPs shared with T2D, and 70% SNPs shared with CAD (Table 2; Supplementary Tables 17–24). The genetic correlations were insignificant (*r*_g_ = −0.06–0.04) (Table 2).

**Table 2:** Shared loci between bipolar disorder and cardiovascular disease phenotypes.

Associated phenotype	Shared loci (n) conjFDR	Concordant effect (%)	Genetic correlation
*CVD phenotypes*			
BMI^a^	69	52.2%	−0.06 (*p* = 0.010)
SBP	53	49.1%	0.02 (*p* = 0.396)
DBP	53	47.2%	0.04 (*p* = 0.155)
TC	15	26.7%	0.02 (*p* = 0.463)
LDL	13	46.2%	0.03 (*p* = 0.346)
HDL	10	40.0%	−0.02 (*p* = 0.471)
T2D	4	25.0%	−0.04 (*p* = 0.422)
CAD	10	70.0%	−0.02 (*p* = 0.539)

Number of shared loci at conjFDR<0.05, concordant effect directions in percentage, and genetic correlation estimated by LD score regression.

*BIP* bipolar disorder, *CVD* cardiovascular disease, *SBP* systolic blood pressure, *DBP* diastolic blood pressure, *BMI* body mass index, *TC* total cholesterol, *HDL* high-density lipoprotein cholesterol, *LDL* low-density lipoprotein cholesterol, T*2D* type 2 diabetes mellitus, *CAD* coronary heart disease, *conjFDR* conjunctional FDR.

^a^The results for BMI & BIP are retrieved from Bahrami et al. [24].

### Functional annotation

Functional annotation of all SNPs having a conjFDR value <0.1 in the loci shared between BIP and CVD phenotypes demonstrated that these were mostly intronic and intergenic (Supplementary Tables 26–33). Gene-mapping of shared loci between BIP and CVD phenotypes largely implicated brain-expressed genes (Supplementary Tables 34–41; Supplementary Results). The initial gene-set analyses of the mapped genes indicated that associations from gene clusters, especially the histone gene cluster, drive many of the significant biological and cellular processes (Supplementary Tables 42–49). Since several genes in these gene sets are localized in a single cluster, a single association in this cluster can drive the apparent enrichment of the entire gene set. Thus, this method is vulnerable to bias from clusters of genes. Therefore, we also performed gene-set analyses of the genes nearest to the lead SNPs in the shared loci between BIP and each CVD phenotype (Supplementary Tables 50–54), and compared the results with the initial analyses. Below, results from both gene-set approaches are presented (i.e., focusing on mapped vs. nearest genes).

Gene-set analyses of mapped genes: First, gene-set analyses revealed several significantly associated biological and cellular processes with the genes mapped to the shared loci between BIP and BMI, including “chromatin organization”, “chromatin assembly/disassembly”, and “DNA packaging complex” (Supplementary Table 42). The genes mapped to the shared loci between BIP and SBP were most significantly associated with “neurogenesis”, “neuronal differentiation”, and “mitochondrion” (Supplementary Table 43). Gene-set analyses also identified several significantly associated processes with the genes mapped to the shared loci between BIP and DBP, including “chromatin assembly”, “nucleosome organization”, and “DNA backpacking complex” (Supplementary Table 44). The genes mapped to the shared loci between BIP and lipids (TC, HDL, and LDL) were significantly associated with “chromatin assembly/disassembly”, “hyaluronan metabolic process”, and “lipid biosynthetic process” (Supplementary Tables 45–47). The genes mapped to loci shared between BIP and T2D and CAD were most significantly associated with “unsaturated fatty acid biosynthesis” (Supplementary Tables 48–49).

The genes nearest to lead SNPs in the shared loci with BMI were significantly associated with “regulation of neurotransmitter levels” and “synapse” (Supplementary Table 50). The genes nearest to lead SNPs in the shared loci between BIP and SBP were most significantly associated with “transmembrane transport”, “regulation of transport”, “plasma membrane region”, “voltage-gated calcium channel complex”, and “synaptic membrane” (Supplementary Tables 51). Gene-set analysis of the genes nearest to lead SNPs in the shared loci between BIP and DBP implicated several biological processes, most significantly “positive regulation of gene expression” and “maintenance of protein localization” (Supplementary Table 52). The genes nearest to lead SNPs in the shared loci between BIP and lipids (TC and LDL) were associated with “chylomicron”, “triglyceride-rich lipoprotein particle” and “protein-lipid complex” (Supplementary Tables 53–54). Finally, there were no significant results from gene-set analysis of genes nearest to lead SNPs in the shared loci between BIP and HDL, T2D and CAD, possibly related to fewer shared loci. In summary, the two approaches to gene-set analysis provided mixed results. Still, they both implicated gene-sets partly associated with neurodevelopment, neurotransmission, lipids, metabolic processes, and regulation of gene expression.

We identified several pathways overrepresented among the genes mapped to loci shared between BIP and CVD phenotypes. We found neural cell adhesion molecule (NCAM) signaling for neurite out-growth pathway to be significantly overrepresented among the genes mapped to the shared loci between BIP and BMI (Supplementary Table 55). Other pathways (e.g., Organelle biogenesis and maintenance, Oxytocin signaling pathway, and Cushing syndrome) were also overrepresented among these genes, but they did not reach significance after correcting for multiple testing (see *q*-values in Supplementary Table 55). We also identified several pathways overrepresented among the genes mapped to loci shared between BIP and SBP/DBP, including signaling by plasma membrane FGR1 fusions, beta-agonist/beta-blocker pathway, sympathetic nerve pathway, cortisol synthesis and secretion, and several hormonal and metabolic pathways (Supplementary Tables 56–57). We found omega-3 fatty acid metabolism pathway and other pathways to be overrepresented among the genes mapped to the shared loci between BIP and lipids, T2D and CAD (Supplementary Tables 58–62).

Spatio-temporal analysis indicated that mapped genes for BIP and CVD phenotypes were expressed in different brain tissues, with increased levels of expression mostly before early adulthood (Supplementary Figure 8a-h). The mapped genes associated with BIP and SBP also exhibited relative upregulation during early adulthood (Supplementary Figure 8b).

## Discussion

In the present study, we demonstrated extensive polygenic overlap between BIP and CVD phenotypes. We revealed 129 shared loci, of which 22 were novel BIP loci. The shared loci possessed mixed effect directions in BIP and CVD risk factors and CAD, consistent with insignificant genetic correlations. The results provide new insights into the shared genetic architecture of BIP and CVD morbidity, implicating novel molecular genetic mechanisms, and may suggest variation in CVD risk across subgroups of patients with BIP.

The present study goes beyond standard methods to assess genetic overlap as the MiXeR can estimate polygenic overlap with mixed effect directions, and the conjFDR method can detect shared genetic variants between phenotypes regardless of the overall genetic correlation [26–28]. Using MiXeR we discovered that ~82% of the genetic variants influencing BIP also influence BMI. In addition, ~20% of variants influencing BIP appear to influence SBP/DBP, yet a larger proportion (~40%) of genetic variants underlying SBP/DBP affect BIP. MiXeR also suggested polygenic overlap between BIP and CAD, although the degree of overlap is uncertain, suggesting that a larger CAD GWAS is needed to obtain more reliable MiXeR estimates. The differences in overlap partly reflect variation in polygenicity of these phenotypes, with BIP and BMI being more polygenic than SBP/DBP and CAD, as illustrated in the Venn diagrams.

Further, conjFDR revealed several shared loci between BIP and CVD phenotypes. More specifically, we identified a total of 227 overlapping loci between BIP and CVD phenotypes at conjFDR<0.05, of which 129 were distinct (Table 2). Most of the loci were shared with BMI [24] and SBP/DBP, while a smaller number of loci were shared with lipids, CAD, and T2D based on conjFDR. While the GWAS sample sizes do not influence the nature of the joint association between BIP and the CVD phenotypes, they are likely to influence the magnitude of genetic overlap across the CVD phenotypes [28]. Accordingly, while the finding of most shared loci with BMI and SBP/DBP suggests greater overlap, this finding may also be related to the larger GWAS samples used for BMI [17] and SBP/DBP [20] than for the other CVD phenotypes [18, 19, 21]. In addition, the highly polygenic nature of BMI likely contributed to the finding of more shared loci with BMI. Interestingly, there was a general pattern of bidirectional effects of the shared loci. Genetic variants with mixed effect directions “cancel each other out”, resulting in insignificant genetic correlations between BIP and CVD risk factors, as well as CAD. Thus, while our results suggest shared molecular mechanisms implicating pleiotropy, there is no clear pattern of increased or decreased genetic liability to CVD in BIP.

The mixed effect directions among the loci shared between BIP and CVD phenotypes underscore the complexity of the genetic relationship. While there was a general trend of opposite effect direction (~52%), there was a majority of concordant effect directions in CAD. However, due to the small number of SNPs involved, these individual loci explain a little proportion of the overall risk. Thus, the findings indicate that common genetic variants do not explain the higher CVD risk in BIP. It is possible that genetic factors not captured by currently GWASs, such as rare variants, may contribute. However, it is likely that environmental risk factors play a central role in comorbid CVD in BIP. In particular, medication, poor nutrition, physical inactivity, and smoking are important contributors to CVD in BIP [8, 45]. The mixed effect directions of the shared loci comply with previous findings of bidirectional effects among overlapping loci between SCZ and multiple CVD risk factors [12]. Similar to the current study, other studies also indicate genetic overlap between BIP and CVD in spite of non-significant genetic correlations [23, 46]. Further, the bidirectional effects of the shared variants between BIP and BMI are in line with the large clinical variation in weight changes during mood episodes of BIP. Some patients experience weight loss while others gain weight during a depressive episode, and most patients lose weight during a manic episode [47]. Similarly, studies suggest variation in lipid levels and SBP/DBP related to affective episodes, with higher levels of dyslipidemia and SBP/DBP in depressive than in manic episodes [48–50].

Further, the mixed effect directions of shared variants may reflect variation in genetic liability to CVD across BIP subgroups. BIP is a heterogeneous disorder involving different subtypes, illness courses, and severity [51] that may be differentially related to CVD comorbidity. Notably, while the average level of CVD risk is higher in BIP compared to the general population, the CVD comorbidity seems to be restricted to BIP subgroups, illustrated by overweight (~50–75%), dyslipidemia (~25–40%), T2D (~5–20%), and hypertension (~35–60%) [2–4], which suggest subsets of patients with different susceptibility to CVD. For instance, patients with more depressive symptoms may represent such a subgroup, as increased depressive symptoms rather than mania are associated with higher rates of obesity, dyslipidemia, and T2D [48–50, 52–55]. Moreover, recent findings indicate a genetic susceptibility to weight gain in major depression [24]. Since BIP type 2 is genetically more related to major depression [30], this subtype of BIP may also involve increased genetic risk of weight gain. BIP type 1, on the other hand, is more genetically correlated with SCZ [30] and may thus have reduced genetic risk of weight gain [24]. Larger and well-characterized GWAS samples are needed to identify subgroups with varied genetic susceptibility to weight gain and other CVD phenotypes in BIP. The identification of potential subgroups with different genetic liability to CVD can increase the understanding of CVD comorbidity in BIP and help improve risk prediction and prevention.

Functional annotation indicated that the shared variants between BIP and CVD are mostly intronic and intergenic, which is in line with other GWAS findings [24, 31]. The results indicate the shared SNPs influence gene expression via regulatory effects [56]. Further functional analyses indicated that the shared variants between BIP and CVD phenotypes are involved in several biological processes and pathways associated with neurodevelopment, lipid metabolism, intracellular processes, and chromatin assembly/disassembly (i.e., formation or destruction of chromatin structures, which play an important role in regulating transcription and gene expression [57]). Further, the shared loci were largely linked to genes expressed in the brain. Spatio-temporal analysis indicated that brain-related genes were mainly expressed prior to early adulthood, which is consistent with the typical BIP onset in late adolescence and early adulthood [58]. However, due to a relatively low number of identified expressed genes, there may be reduced statistical power with risk of false-negative findings. This limits our ability to draw inferences about expression in particular developmental periods and brain regions. In line with current findings, brain dysfunction is implicated in the pathophysiology of BIP [59] and more recently linked to the shared variants between BIP and BMI [22, 24]. Moreover, lipid biology may be involved in the pathophysiology of BIP, as proposed for SCZ [12], consistent with evidence of white-matter abnormalities and myelin dysfunction in both disorders [60, 61]. Furthermore, functional analyses of the shared loci between BIP and SBP implicated genes involved in stress-related pathways, including cortisol synthesis and secretion. Similarly, recent findings indicate overlapping genetic variants between BIP and CVD risk factors associated with hypothalamic–pituitary–adrenal (HPA) axis regulation [22, 24]. Shared genetic variants associated with the HPA axis appear plausible given evidence of HPA axis dysregulation in BIP [62], obesity, and hypertension [63]. However, the results from functional analyses should be considered with caution given the limitations of ConsensusPathDB and FUMA, including vulnerability to bias from clusters of genes in the genome. This bias was evident in the results from gene-set analyses of the shared loci between BIP and CVD phenotypes, indicating that some of the most significant biological processes are driven by associations from the histone gene cluster and other clusters.

Altogether, the current findings are in line with the hypothesis that brain-related mechanisms play a role in CVD comorbidity in BIP. It is possible that shared genetic variants, interacting with environmental risk factors, affect brain function that influences behavior (e.g., lifestyle choices) and mental processes (e.g., affective symptoms) and, thereby, the development of BIP and comorbid CVD. It is also possible that shared variants between BIP and CVD morbidity affect metabolic mechanisms [22, 24], influencing CVD risk and brain function, contributing to the development of BIP. In addition, separate pathways underlying BIP and CVD are likely given the bidirectional effects of the shared loci. However, the proposed pathways are preliminary and require further experimental investigation due to limitations of current methods used to functionally annotate SNPs [33] and the complexity of the pathophysiology of BIP and CVD.

The current results of mixed effect directions of shared loci between BIP and CVD phenotypes have important clinical implications. The results indicate that the genetic susceptibility for CVD may vary across BIP subgroups, calling for more diverse and targeted clinical interventions. Future investigations of subgroups with different genetic liability to CVD can form the basis for improved prediction tools, which can pave the way for early risk identification and prevention of CVD in BIP. Improved prevention should involve better tailored pharmacological treatment according to individuals’ genetic risk and personalized lifestyle interventions with focus on the barriers for maintaining a healthy lifestyle, such as motivational and other affective symptoms, adverse effects of medication, and socioeconomic issues [64, 65].

In conclusion, the current study revealed polygenic overlap between BIP and CVD phenotypes and identified 129 shared loci with mixed effect directions. Future experimental studies of the identified shared loci may provide new insights into molecular mechanisms, which can ultimately facilitate the development of drugs with less cardiometabolic adverse effects by identifying potential therapeutic targets. The current results underline the importance of environmental factors in development of CVD comorbidity in BIP and may indicate variation in genetic susceptibility to CVD across BIP subgroups. Future studies with larger GWAS samples should focus on identifying patients at higher genetic risk of comorbid CVD. This can form the basis for risk stratification and more targeted interventions for better prevention of CVD in BIP.

## Supplementary information

Supplementary tables 1-62

Supplementary information

## Data Availability

The datasets analyzed during the current study are available in repositories of GWASs: BIP: https://www.med.unc.edu/pgc/download-results/bip/; BMI: https://portals.broadinstitute.org/collaboration/giant/index.php/GIANT_consortium_data_files; TC, LDL, and HDL: http://csg.sph.umich.edu/abecasis/public/lipids2013/; T2D: https://diagram-consortium.org/downloads.html; SBP and DBP: http://ldsc.broadinstitute.org/ldhub/; CAD: http://www.cardiogramplusc4d.org/data-downloads/.

## Code availability

Codes used for carrying out the described analyses are available here: https://github.com/precimed/pleiofdr; https://github.com/precimed/mixer; https://github.com/bulik/ldsc. All data/results generated during the current study are included in this published article [and its supplementary information files]. Supplementary information is available at the journal’s website.
