# Supplementary material for: Extensive bidirectional genetic overlap between bipolar disorder and cardiovascular disease phenotypes
Source: Transl Psychiatry. 2021 Jul 23;11:407. doi: 10.1038/s41398-021-01527-z (PMC8302675; doi:10.1038/s41398-021-01527-z)
Supplement: Supplementary file 2 — Supplementary information [file 41398_2021_1527_MOESM2_ESM.pdf]

SUPPLEMENTARY INFORMATION for

Extensive bidirectional genetic overlap between bipolar disorder and cardiovascular disease phenotypes

SUPPLEMENTARY METHODS

Participants

We obtained GWAS results in the form of summary statistics (p-values and z-scores). Data on bipolar disorder (BIP) were retrieved from Psychiatric Genomics Consortium (PGC)[1]. The BIP dataset consisted of 20 352 cases and 31 358 controls from 32 samples[1]. Among the cases, 14,879 individuals were diagnosed with BIP type I (BIP1), 3,421 with BIP type II (BIP2), 977 with schizoaffective disorder, bipolar type (SAB), and the remaining BIP not otherwise specified (NOS)[1]. Further, we used data from GWASs on cardiovascular disease (CVD) phenotypes, including the CVD risk factors body mass index[2] (n=795 640), type 2 diabetes mellitus (T2D)[3] (n=159 208), total cholesterol (TC)[4] (n=188 578), low-density lipoprotein (LDL) cholesterol[4] (n=188 578), high-density lipoprotein (HDL) cholesterol[4] (n=188 578), systolic and diastolic blood pressure (n=745 820-757 601)[5], along with coronary artery disease (CAD, n=332 477, including 71 602 CAD cases and 260 875 controls)[6]. We repeated the previously published cond/conjFDR analysis of genetic overlap between BIP and BMI[7]. Details about the inclusion criteria, genotyping and phenotype characteristics, see the original publications[1-6]. There was no sample overlap between the BIP GWAS[1] and the CVD phenotype GWASs.

MiXeR

We applied causal mixture models[8, 9] to the GWAS summary statistics, using the MiXeR tool (https://github.com/precimed/mixer). For each SNP, i, univariate MiXeR models its additive

genetic effect of allele substitution, β_i, as a point-normal mixture, $\beta_i = (1-\pi_1)N(0,0) + \pi_1 N(0, \sigma_\beta^2)$, where π_1 represents the proportion of non-null SNPs (`polygenicity`) and σ_β^2 represents variance of effect sizes of non-null SNPs (`discoverability`). Then, for each SNP, j, MiXeR incorporates LD information and allele frequencies for M=9,997,231 SNPs extracted from 1000 Genomes Phase3 data by LD score regression software[9, 10], and estimate the expected probability distribution of the signed test statistic, $z_j = \delta_j + \epsilon_j = N \sum_i \sqrt{H_i} r_{ij} \beta_i + \epsilon_j$, where N is sample size, H_i indicates heterozygosity of i-th SNP, r_{ij} indicates allelic correlation between i-th and j-th SNPs, and $\epsilon_j \sim N(0, \sigma_0^2)$ is the residual variance. Further, the three parameters, $\pi_1, \sigma_\beta^2, \sigma_0^2$, are fitted by direct maximization of the likelihood function. The number of trait-influencing variants (i.e. variants with pure genetic effects not induced by LD) is estimated as $M\pi_1$, where M=9,997,231 gives the number of SNPs in the reference panel.

In the cross-trait analysis, MiXeR models additive genetic effects as a mixture of four components, representing null SNPs in both traits (π_0); SNPs with a specific effect on the first and on the second trait (π_1 and π_2, respectively); and SNPs with non-zero effect on both traits (π_{12}). In the last component, MiXeR models variance-covariance matrix as $\mathbf{\Sigma_{12}} = \begin{bmatrix} \sigma_1^2 & \rho_{12}\sigma_1\sigma_2 \\ \rho_{12}\sigma_1\sigma_2 & \sigma_2^2 \end{bmatrix}$ where ρ_{12} indicates correlation of effect sizes within the shared component, and σ_1^2 and σ_2^2 correspond to the discoverability parameter estimated in the univariate analysis of the two traits. After fitting parameters of the model, genetic correlation is calculated as $r_g = \frac{\rho_{12}\pi_{12}}{\sqrt{(\pi_1+\pi_{12})(\pi_2+\pi_{12})}}$. Further information is available in[8].

To evaluate model fit, i.e. the ability of the MiXeR to predict the actual GWAS data, we constructed modelled vs. actual conditional Q-Q plots (Supplementary Figures 3-6). Optimal model fit is indicated in the conditional Q-Q plots by the model-based curves closely following the actual Q-Q curves[8]. Model fit was also assessed using negative log-likelihood

plots[8], which visualizes the performance of the best model versus models with minimum and maximum polygenic overlap (Supplementary Figures 3-6). The best model represents the MiXeR model of polygenic overlap between phenotypes. The minimum model represents a scenario of least possible overlap, and the maximum model represents a scenario of largest possible overlap. In the negative log-likelihood plot (Supplementary Figures 3-6), the minimum model is represented by the point furthest to the left, the maximum model is represented by the point furthest to the right, and the best model is represented by the lower point of the curve. The lowest point on the curve (y-axis) indicates better model fit[8].

To filter situations with insufficiently powered GWAS summary statistics, we use Akaike information criterion ($AIC = 2k - 2\ln L$), where k is the number of free parameters in the model, L is the value of the likelihood function, and n is the effective number of SNPs used in optimization procedure. We calculate the difference between *AIC* for the full bivariate model, $k = 3$, and AIC for the reduced bivariate model, $k = 2$, due to π_{12} being constrained to smallest or largest possible ($\pi_{12}^{min} = r_g\sqrt{\pi_1^u\,\pi_2^u}$ and $\pi_{12}^{max} = \min(\pi_1^u, \pi_2^u)$, respectively). A positive value of AIC indicates that GWAS summary statistics have enough information to distinguish the custom polygenic overlap, as shown on the MiXeR Venn diagrams, from the constrained models with minimal (π_{12}^{min}) and maximum (π_{12}^{max}) polygenic overlap.

Conditional False Discovery Rate

The ‘enrichment’ seen in the conditional Q-Q plots can be directly interpreted in terms of true discovery rate (TDR = 1 – false discovery rate (FDR))[11]. More specifically, for a given p-value cutoff, the FDR is defined as

$$\mathrm{FDR}(p) = \pi_0 F_0(p) / F(p), \qquad [1]$$

where π_0 is the proportion of null SNPs, F_0 is the null cumulative distribution function (cdf), and F is the cdf of all SNPs, both null and non-null[12]. Under the null hypothesis, F_0 is the cdf

of the uniform distribution on the unit interval [0,1], so that Eq. [1] reduces to

$$FDR(p) = \pi_0 p / F(p), \qquad [2]$$

The cdf F can be estimated by the empirical cdf $q = N_p / N$, where N_p is the number of SNPs with p-values $\leq p$, and N is the total number of SNPs. Replacing F by q in Eq. [2], we get

$$\text{Estimated } FDR(p) = \pi_0 p / q, \qquad [3]$$

which is biased upwards as an estimate of the FDR[13]. Replacing π_0 in Equation [3] with unity gives an estimated FDR that is further biased upward;

$$q^* = p / q, \qquad [4]$$

If π_0 is close to one, which is probably true for most GWASs, the increase in bias from Eq. [3] is minimal. Therefore, the quantity 1 – p/q, is biased downward and thus a conservative estimate of the TDR. Referring to the Q-Q plots, we see that q* is equivalent to the nominal p-value divided by the empirical quantile, as defined previously. We can thus read the FDR estimate directly off the Q-Q plot as

$$-\log_{10}(q^*) = \log_{10}(q) - \log_{10}(p), \qquad [5]$$

demonstrating that the estimated FDR is directly related to the horizontal shift of the curves in the Q-Q plots from the expected line x = y, i.e. a larger shift corresponds to a smaller FDR.

Conditional Q-Q plots

Q-Q plots compare a nominal probability distribution against an empirical distribution. In the presence of all null relationships, nominal p-values form a straight line on a Q-Q plot when plotted against the empirical distribution. For BIP and CVD phenotype SNPs and for each categorical subset (strata), $-\log_{10}$ nominal p-values were plotted against $-\log_{10}$ empirical p-values (conditional Q-Q plots). Leftward deflections of the observed distribution from the projected null line illustrate increased tail probabilities in the distribution of test statistics (z-scores) and consequently an over-abundance of low p-values compared to that expected by

chance, also called ‘enrichment’. This is illustrated in Supplementary Figures 1-2.

Under large-scale testing paradigms, such as GWAS, we can calculate quantitative estimates of likely true associations from the distributions of summary statistics[12, 14]. Conditional Q-Q plots of nominal p-values from GWAS summary statistics visualizes this enrichment of statistical association relative to that expected under the global null hypothesis. The usual Q-Q curve has the nominal p value, denoted by "p", as the y-ordinate and the corresponding value of the empirical cdf, denoted by "q", as the x-ordinate. Under the global null hypothesis the theoretical distribution is uniform on the interval [0,1]. As is common in GWAS, we instead plot $-\log_{10}$ p against $-\log_{10}$ q to emphasize tail probabilities of the theoretical and empirical distributions. Therefore, genetic enrichment is illustrated with a leftward shift in the Q-Q curve, corresponding to a larger fraction of SNPs with nominal $-\log_{10}$ p-value greater than or equal to a given threshold. Conditional Q-Q plots are constructed by creating subsets of SNPs based on levels of an auxiliary measure for each SNP, and computing Q-Q plots separately for each level. If SNP enrichment is captured by variation in the auxiliary measure, which is expressed as successive leftward deflections in a conditional Q-Q plot as levels of the auxiliary measure increase. We constructed conditional Q-Q plots of empirical quantiles of nominal $-\log_{10}$ values for SNP association for all SNPs, and for subsets (strata) of SNPs determined by the nominal p-values of their association with the conditional phenotypes, and vice versa. In particular, we computed the empirical cumulative distribution (cdf) of nominal p-values for a given phenotype for all SNPs and for SNPs with significance levels below the indicated cut-offs for the conditional phenotypes ($-\log_{10}(p) \geq 1$, $-\log_{10}(p) \geq 2$, $-\log_{10}(p) \geq 3$ corresponding to $p < 0.1$, $p < 0.01$, $p < 0.001$ respectively). The nominal p-values ($-\log_{10}(p)$) are plotted on the y-axis, and the empirical quantiles ($-\log_{10}(q)$, where q=1-cdf(p)) are plotted on the x-axis. To assess for polygenic effects below the standard GWAS significance threshold, we focused the conditional Q-Q plots on SNPs with nominal $-\log_{10}(p)$

< 7.3 (corresponding to $p > 5x10^{-8}$). We controlled for spurious enrichment by calculating all conditional Q-Q plots after random pruning averaged over 500 iterations. At each iteration, one SNP in every LD block (defined by an $r^2 > 0.1$) was randomly selected and the empirical cdfs were computed using the corresponding p-values.

Detection of SNPs using conditional and conjunctional FDR

The FDR can be interpreted as the probability that a SNP is null given that its p-value is as small as or smaller than its observed p-value. The conditional FDR (condFDR) is an extension of the standard FDR, which incorporates information from GWAS summary statistics of a second phenotype to adjust its significance level. The condFDR is defined as the probability that a SNP is null in the first phenotype given that the p-values in the first and second phenotypes are as small as or smaller than the observed ones. It is important to note that ranking SNPs by the standard FDR or by p-values gives the same ordering of SNPs. In contrast, ranking SNPs by condFDR will reorder SNPs when the primary and secondary phenotypes are genetically related. The conjunctional FDR (conjFDR) is defined as the posterior probability that a SNP is null for either phenotype or both simultaneously, given that its p-values for association with both phenotypes are as small as or smaller than the observed p-values[15-19]. A conservative estimate of the conjFDR is obtained by the maximum condFDR for a given SNP after repeating the condFDR procedure for both traits and inverting their roles.[20]. Given that complex correlations in regions with intricate LD can bias FDR estimation[21], we excluded SNPs in the extended major histocompatibility complex and chromosome 8p23.1 (genome build 19 locations 25119106–33854733 and 7242715–12483982, respectively) and SNPs in LD ($r^2 > 0.1$) with such SNPs before fitting the FDR models. P-values were corrected for inflation using a genomic inflation control procedure[15].

Genomic loci definition

We defined independent genomic loci using the FUMA, an online tool for functional mapping of genetic variants (http://fuma.ctglab.nl/)[22]. Summary statistics from the GWASs on BIP and CVD phenotypes were used as input for FUMA. First, *independent significant SNPs* were identified as SNPs with condFDR < 0.01 and independent from each other at LD $r^2 < 0.6$. Secondly, *lead SNPs* were identified by retaining those independent significant SNPs that were independent from each other at $r^2 < 0.1$. Next, *distinct genomic loci* were identified by merging physically overlapping lead SNPs (LD blocks < 250 kb apart) selecting a SNPO with the most significant p-value as a lead SNP if the merged locus. Borders of the genomic loci were determined by identifying all SNPs in LD ($r^2 \geqq 0.6$) with one of the independent significant SNPs in the locus. The region containing all of these *candidate SNPs* was regarded as a single independent genomic locus. All LD information was calculated from the 1000 Genomes Project reference panel[23].

Effect sizes and genetic correlation

Effect size (z-scores) of the shared SNPs were obtained from the original summary statistics (see original publications[1, 3, 4, 6, 24]). We estimated the genetic correlation using LD score regression[25]. LD score regression was estimated using the Python-based package available at https://github.com/bulik/ldsc. The procedure is described in the documentation of the package (https://github.com/bulik/ldsc/wiki/Heritability-and-Genetic-Correlation).

Identification of novel BIP loci

We identified novel BIP loci by comparing the identified loci at conjFDR <0.05 with the loci reported in the original BIP GWAS[1], the most recent BIP GWAS[26], the NHGRI-EBI catalog[27], previous cond/conjFDR analyses and other studies reporting genome-wide

significant BIP loci[1, 7, 28-45]. The most recent BIP GWAS[26] was not yet published and the genomewide data were not available for analyses when the current study was performed. Thus, we had to obtain the main results of the BIP GWAS from the preprint version on MedRxiv for comparison with the findings of our current analysis. The main results of the BIP GWAS did not change from preprint to published version[26].

Functional annotation

We used FUMA[22], an online annotation platform (http://fuma.ctglab.nl/) to functionally annotated all candidate SNPs in the genomic loci with a condFDR or conjFDR value<0.10 having an $r^2 \geqq 0.6$ with one of the independent significant SNPs. SNPs were annotated with Combined Annotation Dependent Depletion (CADD) scores[46], RegulomeDB[47] scores, and chromatin states[48, 49]. The CADD score is a deleterious score of variants computed by integrating 63 functional annotations[46]. The higher the score, the more deleterious. A CADD score above 12.37 is the threshold to be potentially pathogenic[46]. The RegulomeDB score is a categorical score to guide interpretation of regulatory variants[47]. It is based on information from eQTLs and chromatin marks, ranging from 1a to 7 with lower scores indicating a higher likelihood of having a regulatory function. Scores are as follows: 1a=eQTL + Transcription Factor (TF) binding + matched TF motif + matched DNase Footprint + DNase peak; 1b=eQTL + TF binding + any motif + DNase Footprint + DNase peak; 1c=eQTL + TF binding + matched TF motif + DNase peak; 1d=eQTL + TF binding + any motif + DNase peak; 1e=eQTL + TF binding + matched TF motif; 1f=eQTL + TF binding / DNase peak; 2a=TF binding + matched TF motif + matched DNase Footprint + DNase peak; 2b=TF binding + any motif + DNase Footprint + DNase peak; 2c=TF binding + matched TF motif + DNase peak; 3a=TF binding + any motif + DNase peak; 3b=TF binding + matched TF motif; 4=TF binding + DNase peak; 5=TF binding or DNase peak; 6=other; 7=Not available[47].

The chromatin state represents the accessibility of genomic regions (every 200bp) with 15 categorical states predicted by a hidden Markov model based on 5 chromatin marks for 127 epigenomes in the Roadmap Epigenomics Project[48]. A lower state indicates increased accessibility, with states 1-7 referring to open chromatin states. We annotated the minimum chromatin state across tissues to SNPs. The 15-core chromatin states as suggested by Roadmap are as follows: 1=Active Transcription Start Site (TSS); 2=Flanking Active TSS; 3=Transcription at gene 5' and 3'; 4=Strong transcription; 5= Weak Transcription; 6=Genic enhancers; 7=Enhancers; 8=Zinc finger genes & repeats; 9=Heterochromatic; 10=Bivalent/Poised TSS; 11=Flanking Bivalent/Poised TSS/Enh; 12=Bivalent Enhancer; 13=Repressed PolyComb; 14=Weak Repressed PolyComb; 15=Quiescent/Low. Standardized SNP effect sizes were calculated for the most impactful SNPs by transforming the sample size-weighted meta-analysis *Z* score, in line with Zhu et al.[49].

Furthermore, using FUMA[22], we linked lead and candidate SNPs to genes applying either of three gene mapping strategies: 1) positional mapping to align SNPs to genes based on their physical proximity (i.e., within a 10kb window), 2) expression quantitative trait locus (eQTL) mapping to match cis-eQTL SNPs to genes whose expression is associated with allelic variation at the SNP level, and 3) chromatin interaction mapping to link SNPs to genes based on three-dimensional DNA–DNA interactions between each SNP's genomic region and nearby or distant genes. We evaluated eleven eQTL databases in FUMA which contains eQTL information from multiple human tissue types including several brain regions (http://fuma.ctglab.nl/tutorial#eQTLs). The eQTL analyses were corrected for multiple comparisons using an FDR threshold of 0.05. FUMA contains Hi-C data of over 21 tissue/cell types including human brain tissue (https://fuma.ctglab.nl/tutorial#chromatin-interactions). We used an FDR of 1×10^{-6} to define significant chromatin interactions based on the suggestion by Schmitt et al.[50]. FUMA was also used to identify previously reported GWAS

associations in the NHGRI-EBI catalog[27] and to evaluate gene ontology (GO)[51] gene-set enrichment for the genes mapped to all (candidate and lead) SNPs in the identified shared loci and genes nearest to lead SNPs in the shared loci at conjFDR<0.05. Finally, we performed pathway over-represented analyses of genes mapped to all (candidate and lead) SNPs in the shared loci using ConsensusPathDB[52]. ConsensusPathDB integrates interaction networks involving binary and complex protein-protein, genetic, metabolic, signaling, gene regulatory and drug-target interactions, along with biochemical pathways[52]. ConsensusPathDB integrates 30 public interaction/pathway resources and has regular content updates, ensuring that this database stays up-to-date and comprehensive[52]. Other GWASs of overlapping loci between complex traits have also applied ConsensusPathDB for pathway analysis[7, 53]. Further, to assess patterns of gene expression in the brain, we performed analysis of mapped genes with reference to RNA-Seq data from Brainspan to selected spatio-temporal gene quantifications at p<0.05 after Bonferroni correction[54-56]. Weighted gene co-expression network analysis (WGCNA) was used to the RNA-Seq dataset from Brainspan to identify pre-determined clusters of co-expressed genes enriched with mapped genes for each phenotypic pair[57]. Spatio-temporal expression heatmaps were generated for the identified clusters[57]. Analyses were corrected for multiple comparisons.

SUPPLEMENTARY RESULTS

MiXeR results

MiXeR results, including number of shared and unique trait-influencing variants and corresponding standard error, are presented in Figure 1 and Table 1. Using MiXeR we discovered extensive polygenic overlap between BIP and BMI, sharing 6.6k out of 12.5k variants involved, as illustrated by the Venn diagram (Figure 1a). The shared variants represent 81.5% of the genetic variants influencing BIP (8.1k) and 60% of the variants

underlying BMI (11.0k). MiXeR also revealed polygenetic overlap between BIP and SBP, sharing 1.8k out of 10.7k variants, as visualized in the Venn diagram (Figure 1b). The shared variants with SBP represent 22.2% of the genetic variants influencing BIP (8.1k), and 40.9% of variants influencing SBP (4.4k). Likewise, MiXeR identified polygenic overlap with DBP, sharing 1.6k out of 10.4K variants, as seen in the Venn diagram (Figure 1c). The shared variants with DBP represent 19.8% of the variants influencing BIP (8.1k) and 41.0% of the variants influencing DBP (3.9k). Finally, using MiXeR we discovered genetic overlap between BIP and CAD, sharing 0.9k out of 8.6k variants, as shown in the Venn diagram (Figure 1d). The overlapping variants constitute 11.1% of the genetic variants influencing BIP (8.1k) and 64.3% of the variants influencing CAD (1.4k).

The MiXeR estimates adequately model the GWAS data, as indicated by the model-based Q-Q plots following the actual Q-Q plots (Supplementary Figures 3-6). However, the model for BIP and CAD followed the actual Q-Q plots less closely at lower p-values (Supplementary Figure 6), suggesting caution in interpreting the data. A larger CAD GWAS is necessary to obtain more reliable MiXeR estimates. The negative log-likelihood plots also illustrated adequate model fit, as indicated by the lowest point on the curve at n=the estimated number of shared variants (Supplementary Figures 3-6). Further, AIC demonstrated sufficient power of the model (Supplementary MiXeR Table). The positive AIC values indicate that the MiXeR model is adequately powered to differentiate the estimated polygenic overlap from minimum possible overlap (best vs. min. overlap) and maximum possible overlap (best vs max. overlap) (Supplementary MiXeR Table).

MiXeR was not applied for the other CVD phenotypes due to inadequate model fit, as demonstrated in the negative log-likelihood plots not showing a clear minimum on the curve (Supplementary Figures 7a-d).

Conditional FDR results

We observed consistent enrichment in BIP conditional on associations with CVD phenotypes (Supplementary Figure 1), and enrichment in CVD phenotypes given associations with BIP (Supplementary Figure 2). This indicates polygenic overlap between BIP and CVD phenotypes. To increase statistical power, we leveraged the pleiotropic enrichment using condFDR analysis and re-ranked BIP SNPs conditional on their association with CVD phenotypes, and vice versa. At condFDR<0.01, we identified 52 loci associated with BIP conditional on their association with BMI (as previously reported[7]); 45 loci conditional on SBP; 42 loci conditional on DBP, 22 conditional on TC, 21 conditional on LDL, 22 conditional on HDL, 32 loci conditional on T2D and 36 loci conditional on CAD (Supplementary Tables 1-8). Next, we identified multiple loci associated with CVD phenotypes conditional on associations with BIP, including 679 loci associated with BMI (as previously reported[7]), 920 loci associated with SBP, 937 loci associated with DBP and 196 loci associated with TC (Supplementary Tables 9-12). Several loci were also associated with LDL (n=147), HDL (n=191), T2D (n=71) and CAD (n=130) conditional on BIP (Supplementary Tables 13-16).

Effect directions of shared lead SNPs between BIP and CVD phenotypes

We evaluated the directionality of allelic effects of the shared lead SNPs between the phenotypes by investigating their z-scores. As denoted by the sign of the effect sizes, there was a pattern of mixed effect directions of the shared SNPs between BIP and CVD risk factors (Table 2). We discovered the same effect direction in 36/69 loci (52%) in BMI and BIP as previously reported[7], 26/53 loci (49.1%) in SBP and BIP, 25/53 loci (47.2%) in DBP and BIP, 4/15 loci (26.7 %) in TC and BIP, 6/13 loci (46.2%) in LDL and BIP, 4/10 loci

(40%) in HDL and BIP, 1/4 loci (25%) in T2D and BIP, and 7/10 loci (70%) in CAD and BIP (Supplementary tables 17-24).

Gene-mapping results

Gene-mapping of *lead* SNPs: Among SNPs shared between BIP and BMI (69), positional mapping aligned the SNPs to 48 genes, cis-eQTL mapping implicated 22 genes, and chromatin interaction mapping implicated no genes (Supplementary Table 17). Among lead SNPs shared with SBP (53), positional mapping linked the SNPs to 42 genes, cis-eQTL mapping indicated 28 genes, and chromatin interaction mapping implicated 4 genes (Supplementary Table 18). Among the SNPs shared with DBP (53), positional mapping linked the SNPs to 42 genes, cis-eQTL mapping linked the SNP to 30 genes, and chromatin interaction mapping implicated 5 genes (Supplementary Table 19). Of SNPs shared with TC (15), positional mapping aligned the SNPs to 13 genes, cis-eQTL mapping implicated 10 genes, and chromatin interaction mapping implicated 2 genes (Supplementary Table 20). Among SNPs shared with LDL (13), positional mapping linked the SNPs to 10 genes, cis-eQTL mapping indicated 8 genes, and chromatin interaction mapping implicated no genes (Supplementary Table 21). Among SNPs shared with HDL (10), positional mapping linked the SNPs to 6 genes, cis-eQTL mapping indicated 8 genes, and chromatin interaction mapping implicated one gene (Supplementary Table 22). Among the SNPs shared with T2D (4), positional mapping linked the SNP to 2 genes, cis-eQTL mapping indicated 3 genes, and chromatin interaction mapping implicated no genes (Supplementary Table 23). Among the 10 SNPs shared with CAD, positional mapping linked the SNP to 6 gene, cis-eQTL mapping indicated 6 genes, and chromatin interaction mapping implicated one gene (Supplementary Table 24). Since chromatin interaction mapping and eQTL mapping were restricted to genes

in the brain, the current results implicated that most of the shared loci were linked to genes expressed in the brain.

Gene-mapping of *candidate* SNPs: Using FUMA, we linked the candidate SNPs in the shared loci between BIP and BMI to 226 protein-coding genes (Supplementary Table 34). Positional mapping linked the SNPs to 159 genes, cis-eQTL mapping linked the SNP to 124 genes, and chromatin interaction mapping implicated 3 genes (Supplementary Table 34). FUMA linked the candidate SNPs in the shared loci between BIP and SBP to 226 protein-coding genes (Supplementary Table 35). Positional mapping linked the SNPs to 159 genes, cis-eQTL mapping indicated 124 genes, and chromatin interaction mapping implicated 3 genes (Supplementary Table 35). FUMA linked the candidate SNPs in the shared loci between BIP and DBP to 282 protein-coding genes (Supplementary Table 36). Positional mapping linked the SNPs to 205 genes, cis-eQTL mapping linked the SNP to 138 genes, and chromatin interaction mapping implicated 20 genes (Supplementary Table 36). FUMA linked the candidate SNPs in the shared between BIP and TC to 109 protein-coding genes (Supplementary Table 37). Positional mapping linked the SNPs to 66 genes, cis-eQTL mapping linked the SNP to 69 genes, and chromatin interaction mapping implicated no genes (Supplementary Table 37). FUMA linked the candidate SNPs in the shared between BIP and LDL to 74 protein-coding genes (Supplementary Table 38). Positional mapping linked the SNPs to 40 genes, cis-eQTL mapping linked the SNP to 53 genes, and chromatin interaction mapping implicated no genes (Supplementary Table 38). FUMA linked the candidate SNPs in the shared between BIP and HDL to 68 protein-coding genes (Supplementary Table 39). Positional mapping linked the SNPs to 35 genes, cis-eQTL mapping linked the SNP to 41 genes, and chromatin interaction mapping implicated 6 genes (Supplementary Table 39). FUMA linked the candidate SNPs in the shared between BIP and T2D to 23 protein-coding genes (Supplementary Table 40). Positional mapping linked the SNPs to 14 genes, cis-eQTL

mapping linked the SNP to 16 genes, and chromatin interaction mapping implicated no genes (Supplementary Table 40). FUMA linked the candidate SNPs in the shared between BIP and CAD to 63 protein-coding genes (Supplementary Table 41). Positional mapping linked the SNPs to 34 genes, cis-eQTL mapping linked the SNP to 44 genes, and chromatin interaction mapping implicated one gene (Supplementary Table 41). In line with the genes mapped to lead SNPs, the majority of the genes mapped to candidate SNPs in the shared loci between BIP and CVD phenotypes were expressed in the brain.

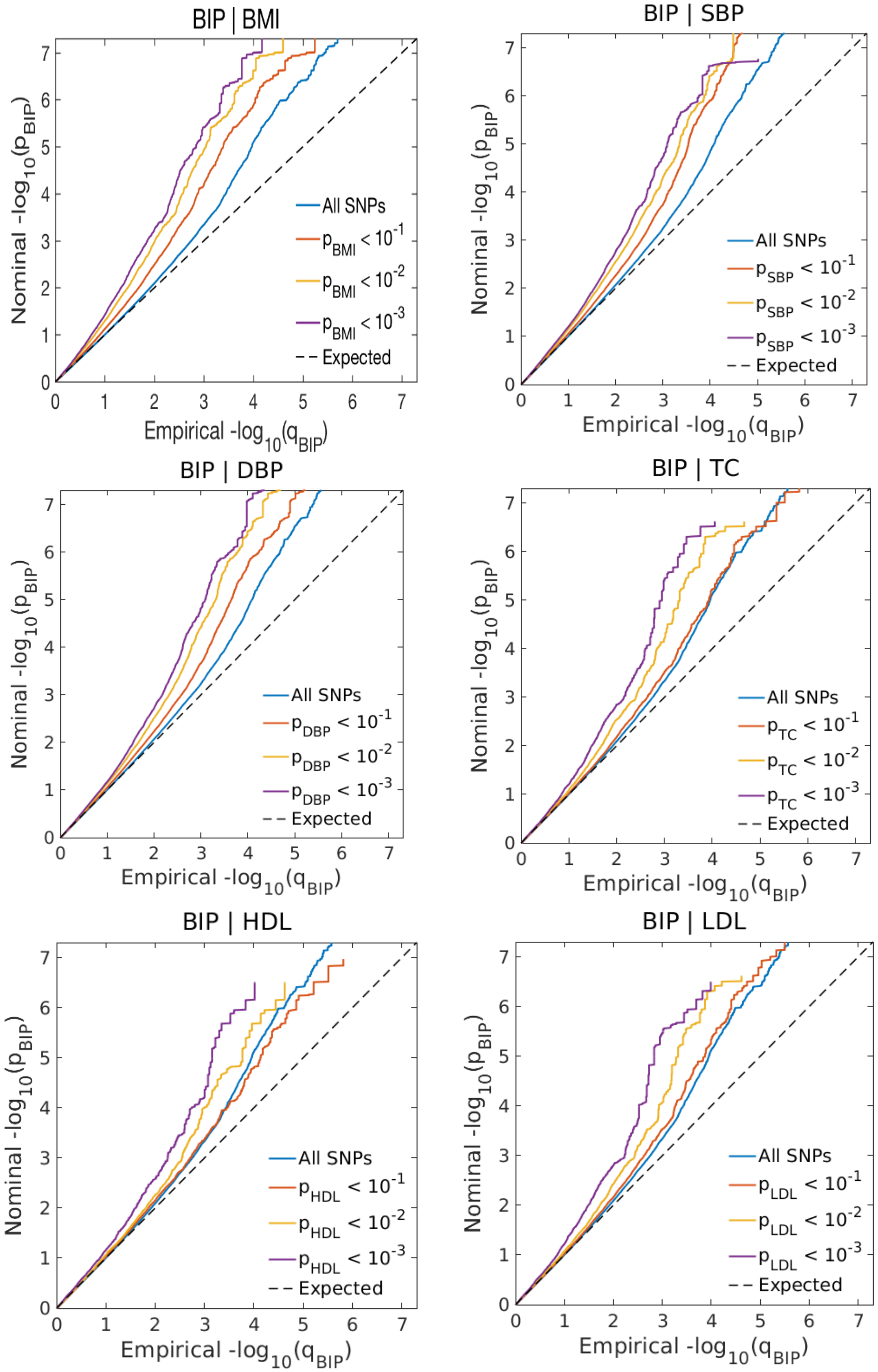

BIP | BMI
Nominal -log10(pBIP)
Empirical -log10(qBIP)
All SNPs
pBMI < 10-1
pBMI < 10-2
pBMI < 10-3
Expected
BIP | SBP
Nominal -log10(pBIP)
Empirical -log10(qBIP)
All SNPs
pSBP < 10-1
pSBP < 10-2
pSBP < 10-3
Expected
BIP | DBP
Nominal -log10(pBIP)
Empirical -log10(qBIP)
All SNPs
pDBP < 10-1
pDBP < 10-2
pDBP < 10-3
Expected
BIP | TC
Nominal -log10(pBIP)
Empirical -log10(qBIP)
All SNPs
pTC < 10-1
pTC < 10-2
pTC < 10-3
Expected
BIP | HDL
Nominal -log10(pBIP)
Empirical -log10(qBIP)
All SNPs
pHDL < 10-1
pHDL < 10-2
pHDL < 10-3
Expected
BIP | LDL
Nominal -log10(pBIP)
Empirical -log10(qBIP)
All SNPs
pLDL < 10-1
pLDL < 10-2
pLDL < 10-3
Expected

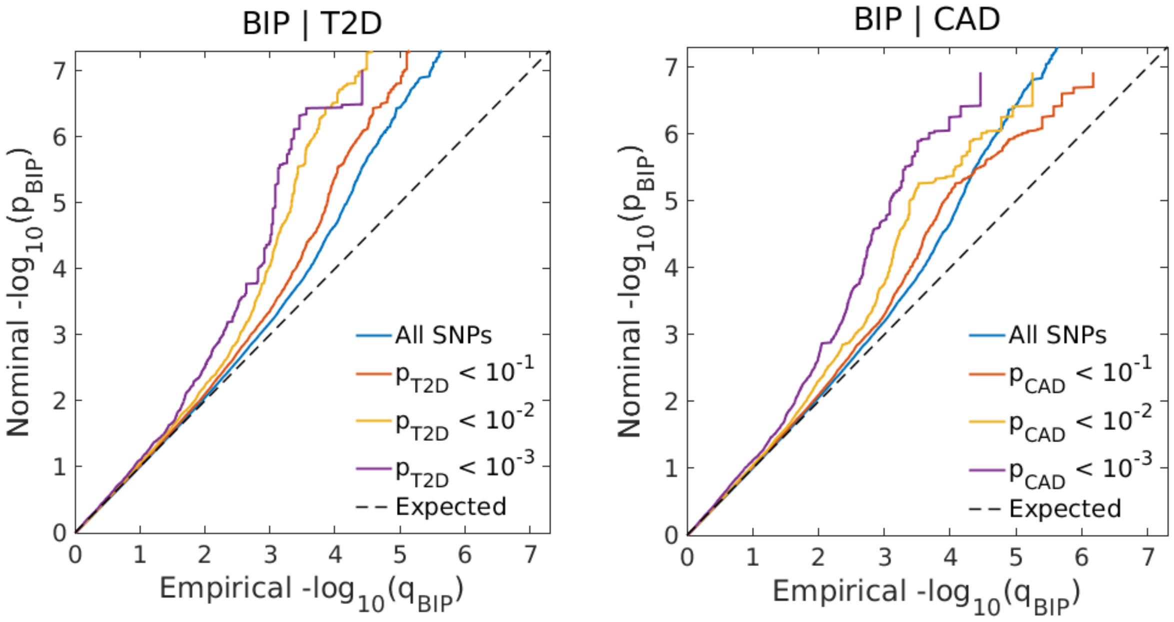

Supplementary Figure 1. Polygenic overlap between BIP and CVD phenotype. Conditional Q-Q plots of nominal versus empirical −log10p values (corrected for inflation) in BIP below the standard GWAS threshold of $p < 5 \times 10{-}8$ as a function of significance of association with CVD phenotype, at the level of $p < 0.1$, $p < 0.01$, $p < 0.001$, respectively. The blue lines indicate all SNPs. The dashed lines indicate the null hypothesis. The Q-Q plot for BIP and BMI is previously published in Bahrami et al. 2020[7]. Abbreviations: BIP, bipolar disorder; CVD, cardiovascular disease; BMI, body mass index; SBP, systolic blood pressure; DBP, diastolic blood pressure; TC, total cholesterol; HDL, high-density lipoprotein cholesterol; LDL, low density lipoprotein cholesterol; T2D, type 2 diabetes; CAD, coronary artery disease. The conditional Q-Q plots build on the condFDR method.

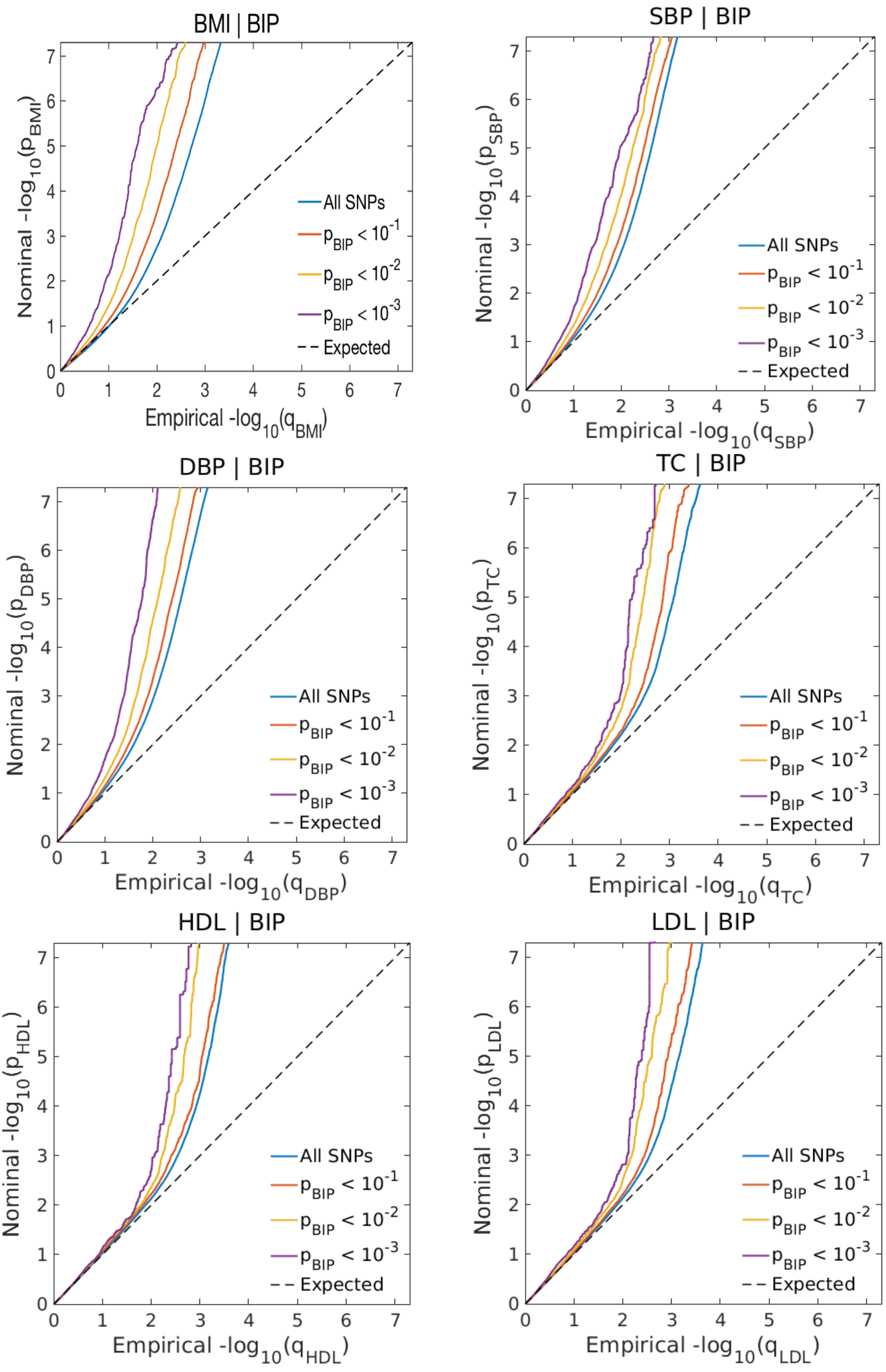
BMI | BIP
Nominal -log10(pBMI)
Empirical -log10(qBMI)
SBP | BIP
Nominal -log10(pSBP)
Empirical -log10(qSBP)
DBP | BIP
Nominal -log10(pDBP)
Empirical -log10(qDBP)
TC | BIP
Nominal -log10(pTC)
Empirical -log10(qTC)
HDL | BIP
Nominal -log10(pHDL)
Empirical -log10(qHDL)
LDL | BIP
Nominal -log10(pLDL)
Empirical -log10(qLDL)
All SNPs
pBIP < 10-1
pBIP < 10-2
pBIP < 10-3
Expected

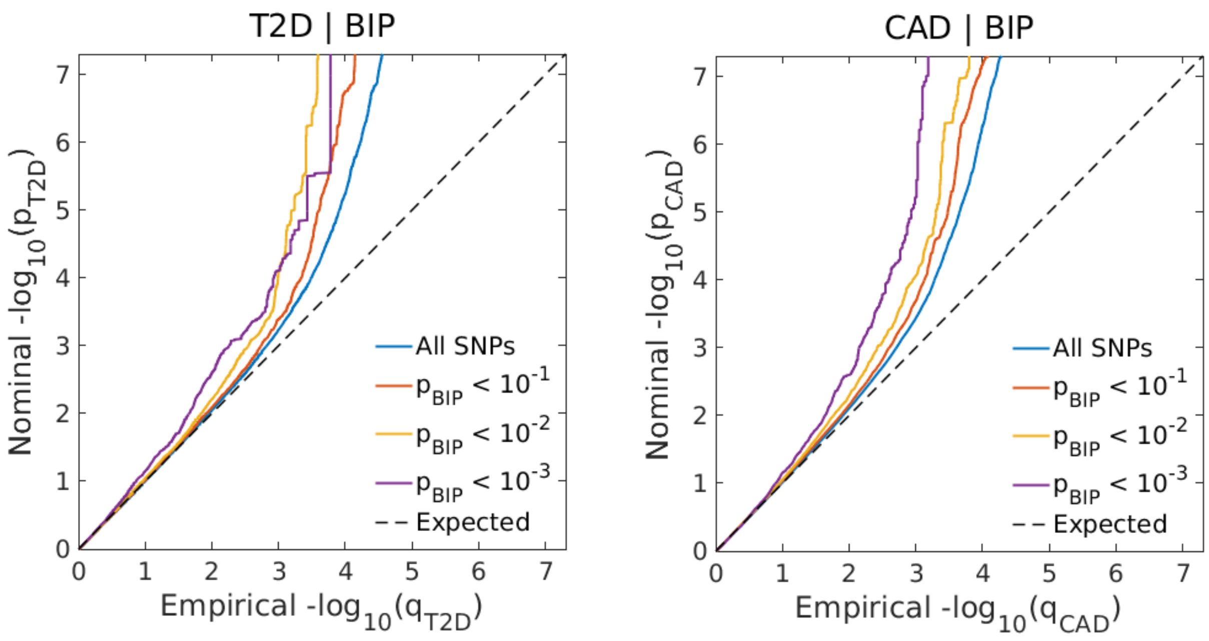

Supplementary Figure 2. Polygenic overlap between BIP and CVD phenotype. Conditional Q-Q plots of nominal versus empirical −log10p values (corrected for inflation) in CVD phenotype below the standard GWAS threshold of $p < 5 \times 10-8$ as a function of significance of association with BIP, at the level of $p < 0.1$, $p < 0.01$, $p < 0.001$, respectively. The blue lines indicate all SNPs. The dashed lines indicate the null hypothesis. The Q-Q plot for BIP and BMI is previously published in Bahrami et al. 2020[7]. Abbreviations: BIP, bipolar disorder; CVD, cardiovascular disease; BMI, body mass index; SBP, systolic blood pressure; DBP, diastolic blood pressure; TC, total cholesterol; HDL, high-density lipoprotein cholesterol; LDL, low density lipoprotein cholesterol; T2D, type 2 diabetes; CAD, coronary artery disease. The conditional Q-Q plots build on the condFDR method.

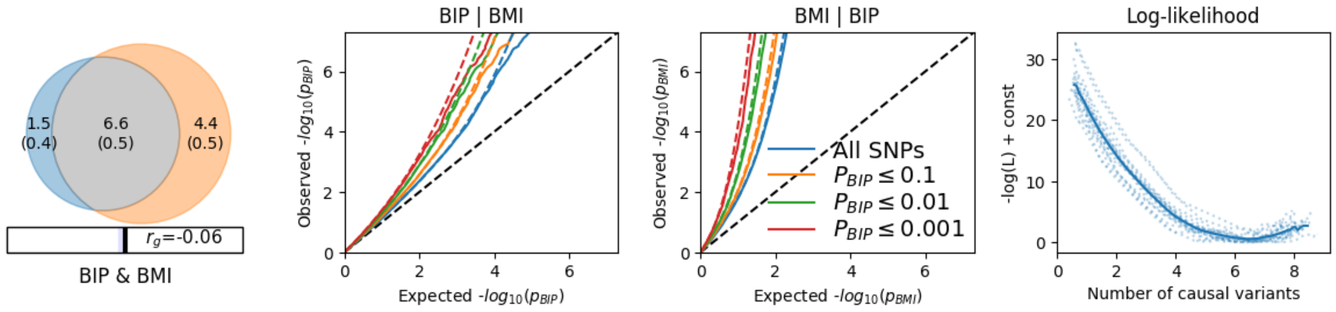

Supplementary Figure 3. Venn Diagrams, conditional Q-Q plots, and negative log-likelihood plot, respectively. *Venn diagrams* of shared and unique trait-influencing variants, showing polygenic overlap (gray) between bipolar disorder (BIP) (blue) and body mass index (BMI) (orange). The numbers in the Venn diagram indicate the estimated quantity of trait-influencing variants (in thousands), explaining 90% of SNP heritability in each phenotype, followed by standard error. *Conditional Q–Q plots* of observed versus expected $-\log_{10}$ *p*-values in the primary trait as a function of significance of association with a secondary trait at the level of $p < 0.1$, $p < 0.01$, $p < 0.001$. Blue line indicates all SNPs. Dotted lines in blue, orange, green, and red indicate model predictions for each stratum. Black dotted line is the expected Q–Q plot under null hypothesis. *Negative log-likelihood plot*: minus log-likelihood calculated for the bivariate model as a function of π parameter. The remaining parameters of the model were constrained to their fitted values. Figure generated from MiXeR.

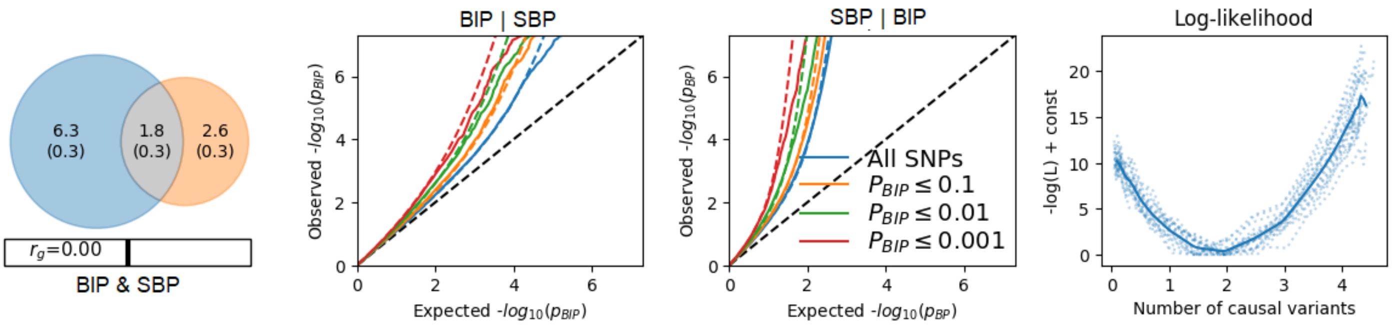

Supplementary Figure 4. Venn Diagrams, conditional Q-Q plots, and negative log-likelihood plot, respectively. *Venn diagrams* of shared and unique trait-influencing variants, showing polygenic overlap (gray) between bipolar disorder (BIP) (blue) and systolic blood pressure (SBP) (orange). The numbers in the Venn diagram indicate the estimated quantity of trait-influencing variants (in thousands), followed by standard error. Appearance of the Q-Q plot and negative log-likelihood plot are described below Supplementary Figure 3. Figure generated from MiXeR.

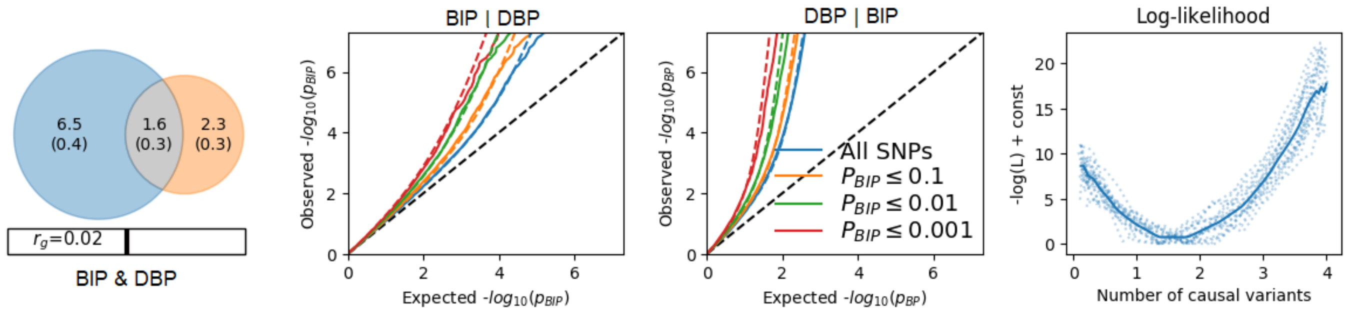

Supplementary Figure 5. Venn Diagrams, conditional Q-Q plots, and negative log-likelihood plot, respectively. *Venn diagrams* of shared and unique trait-influencing variants, showing polygenic overlap (gray) between bipolar disorder (BIP) (blue) and diastolic blood pressure (DBP) (orange). The numbers in the Venn diagram indicate the estimated quantity of trait-influencing variants (in thousands), followed by standard error. Appearance of the Q-Q plot and negative log-likelihood plot are described below Supplementary Figure 3. Figure generated from MiXeR.

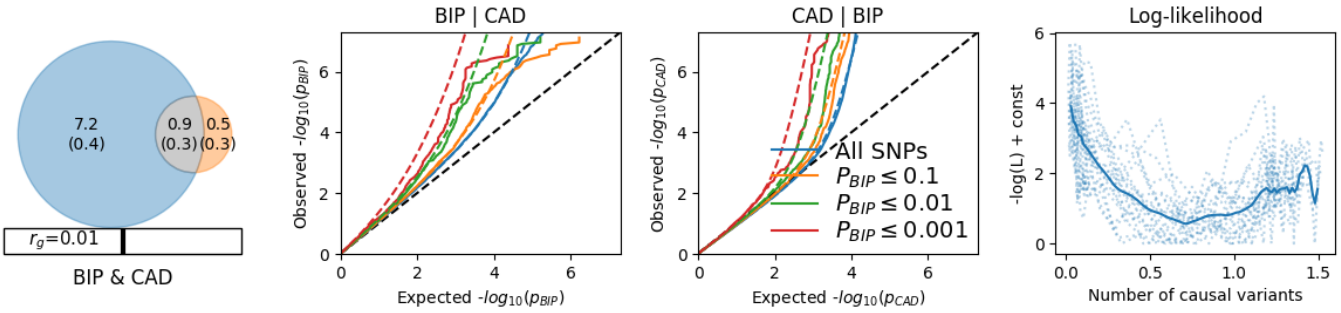

Supplementary Figure 6. Venn Diagrams, conditional Q-Q plots, and negative log-likelihood plot, respectively. *Venn diagrams* of shared and unique trait-influencing variants, showing polygenic overlap (gray) between bipolar disorder (BIP) (blue) and coronary artery disease (CAD) (orange). The numbers in the Venn diagram indicate the estimated quantity of trait-influencing variants (in thousands, followed by standard error. Appearance of the Q-Q plot and negative log-likelihood plot are described below Supplementary Figure 3. Figure generated from MiXeR.

**Supplementary MiXeR Table. Results of cross-trait analysis with the MiXeR model**			
**Trait 1**	**Trait 2**	**AIC**	
		best vs min. overlap	best vs max. overlap
BIP	BMI	52.14	5.69
BIP	SBP	23.21	35.21
BIP	DBP	17.68	36.64
BIP	CAD	6.00	3.69
AIC - results from Akaike information criterion, showing AIC calculated for the full versus reduced bivariate MiXeR model, constrained to minimal feasible polygenic overlap (“best vs min.”) or to the complete polygenic overlap (“best vs max.”). A positive AIC value provides evidence for the polygenic overlap, shown in the MiXeR Venn diagram. BIP: Bipolar disorder; BMI, body mass index; SBP, systolic blood pressure; DBP, diastolic blood pressure; CAD, coronary artery disease.			

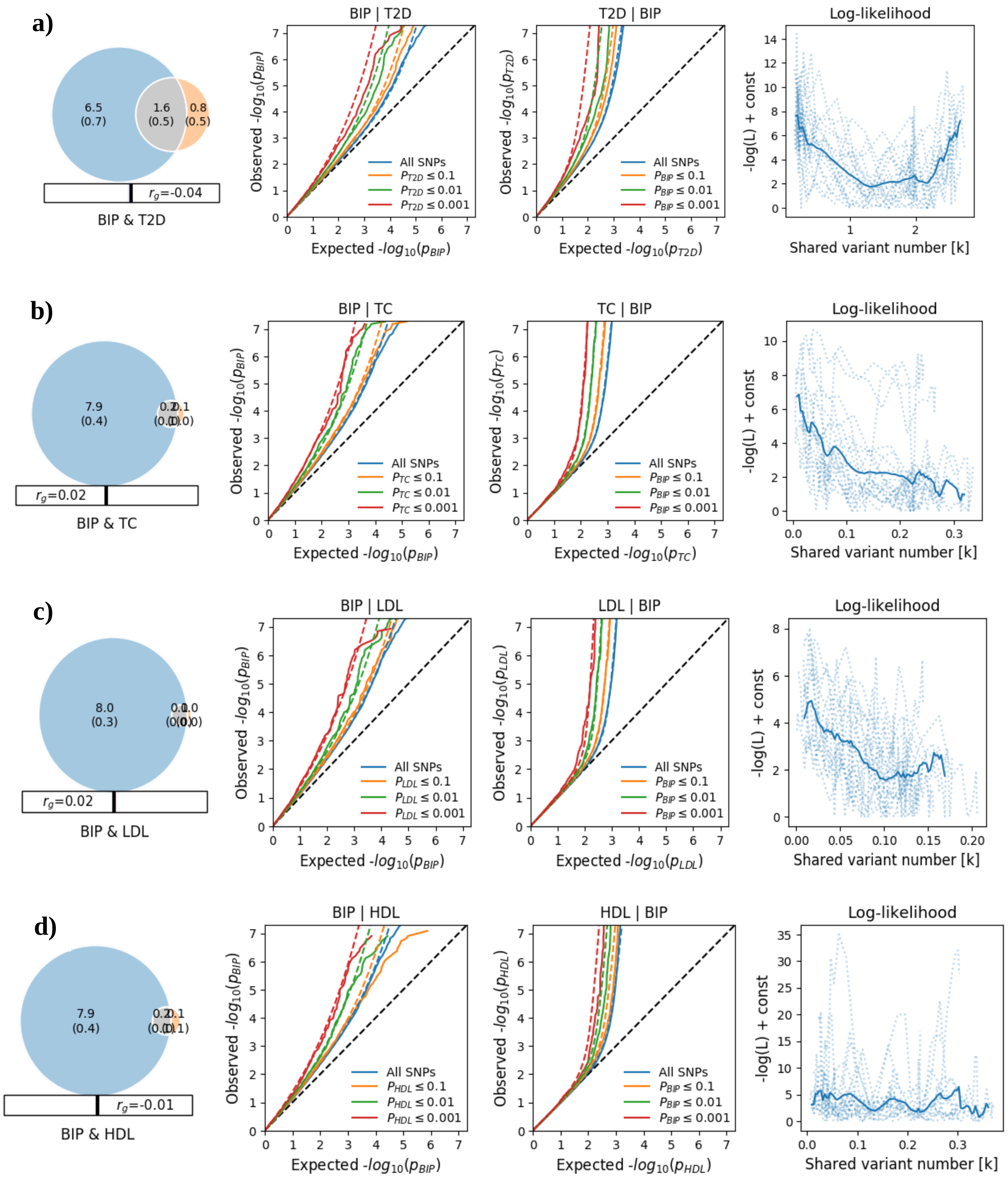
a)
6.5
(0.7)
1.6
(0.5)
0.8
(0.5)
r_g=-0.04
BIP & T2D
BIP | T2D
T2D | BIP
Log-likelihood
Observed -$log_{10}(p_{BIP})$
Expected -$log_{10}(p_{BIP})$
Observed -$log_{10}(p_{T2D})$
Expected -$log_{10}(p_{T2D})$
All SNPs
$P_{T2D} \leq 0.1$
$P_{T2D} \leq 0.01$
$P_{T2D} \leq 0.001$
$P_{BIP} \leq 0.1$
$P_{BIP} \leq 0.01$
$P_{BIP} \leq 0.001$
-log(L) + const
Shared variant number [k]
b)
7.9
(0.4)
r_g=0.02
BIP & TC
BIP | TC
TC | BIP
Log-likelihood
Observed -$log_{10}(p_{TC})$
Expected -$log_{10}(p_{TC})$
$P_{TC} \leq 0.1$
$P_{TC} \leq 0.01$
$P_{TC} \leq 0.001$
c)
8.0
(0.3)
r_g=0.02
BIP & LDL
BIP | LDL
LDL | BIP
Observed -$log_{10}(p_{LDL})$
Expected -$log_{10}(p_{LDL})$
$P_{LDL} \leq 0.1$
$P_{LDL} \leq 0.01$
$P_{LDL} \leq 0.001$
d)
7.9
(0.4)
r_g=-0.01
BIP & HDL
BIP | HDL
HDL | BIP
Observed -$log_{10}(p_{HDL})$
Expected -$log_{10}(p_{HDL})$
$P_{HDL} \leq 0.1$
$P_{HDL} \leq 0.01$
$P_{HDL} \leq 0.001$

Supplementary Figure 7a-d. Venn Diagrams, conditional Q-Q plots, and negative log-likelihood plot, respectively. *Venn diagrams* of shared and unique trait-influencing variants, showing polygenic overlap (gray) between bipolar disorder (BIP) (blue) and **a)** type 2 diabetes (T2D) (orange), **b)** total cholesterol (TC) (orange), **c)** low-density lipoprotein (LDL) (orange) and **d)** high-density lipoprotein (HDL) (orange). The numbers in the Venn diagram indicate the estimated quantity of trait-influencing variants (in thousands), explaining 90% of SNP heritability in each phenotype, followed by standard error. *Conditional Q–Q plots* of observed versus expected $-\log_{10}$ *p*-values in the primary trait as a function of significance of association with a secondary trait at the level of $p < 0.1$, $p < 0.01$, $p < 0.001$. Blue line indicates all SNPs. Dotted lines in blue, orange, green, and red indicate model predictions for each stratum. Black dotted line is the expected Q–Q plot under null hypothesis. *Negative log-likelihood plot*: minus log-likelihood calculated for the bivariate model as a function of π parameter. The remaining parameters of the model were constrained to their fitted values. Figure generated from MiXeR.

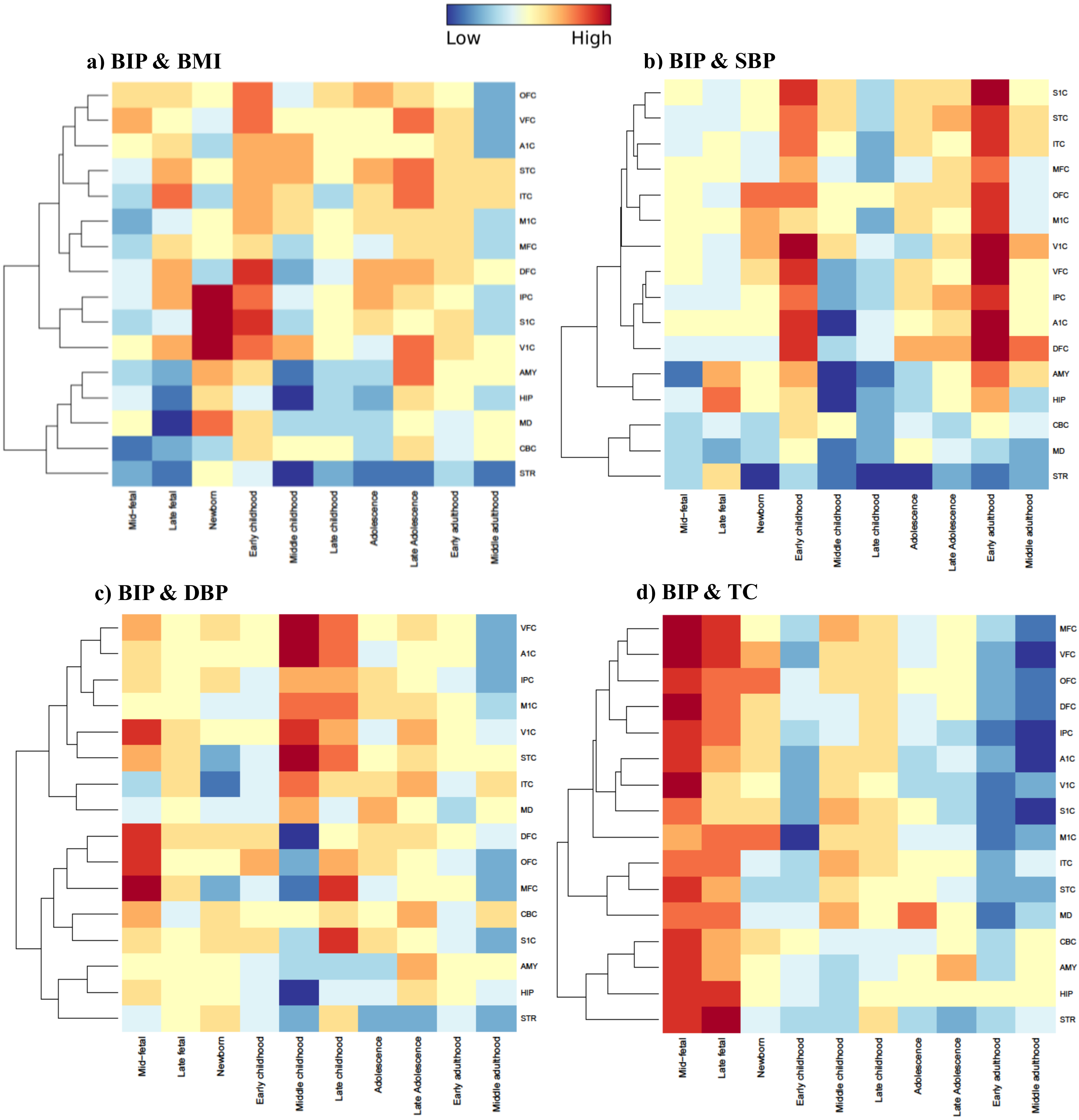
Low
High
a) BIP & BMI
OFC
VFC
A1C
STC
ITC
M1C
MFC
DFC
IPC
S1C
V1C
AMY
HIP
MD
CBC
STR
Mid-fetal
Late fetal
Newborn
Early childhood
Middle childhood
Late childhood
Adolescence
Late Adolescence
Early adulthood
Middle adulthood
b) BIP & SBP
S1C
STC
ITC
MFC
OFC
M1C
V1C
VFC
IPC
A1C
DFC
AMY
HIP
CBC
MD
STR
Mid-fetal
Late fetal
Newborn
Early childhood
Middle childhood
Late childhood
Adolescence
Late Adolescence
Early adulthood
Middle adulthood
c) BIP & DBP
VFC
A1C
IPC
M1C
V1C
STC
ITC
MD
DFC
OFC
MFC
CBC
S1C
AMY
HIP
STR
Mid-fetal
Late fetal
Newborn
Early childhood
Middle childhood
Late childhood
Adolescence
Late Adolescence
Early adulthood
Middle adulthood
d) BIP & TC
MFC
VFC
OFC
DFC
IPC
A1C
V1C
S1C
M1C
ITC
STC
MD
CBC
AMY
HIP
STR
Mid-fetal
Late fetal
Newborn
Early childhood
Middle childhood
Late childhood
Adolescence
Late Adolescence
Early adulthood
Middle adulthood

e) BIP & LDL

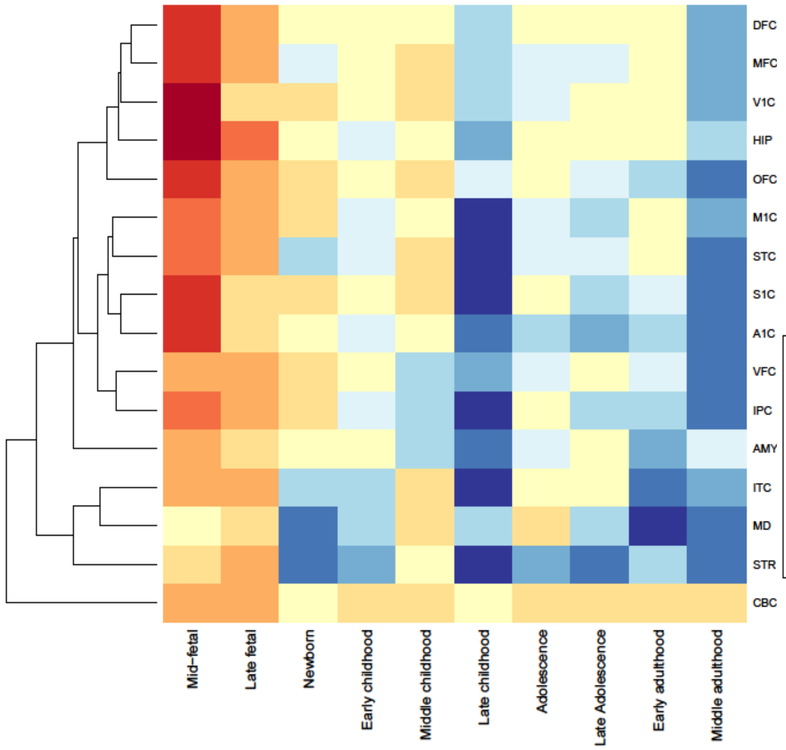

f) BIP & HDL

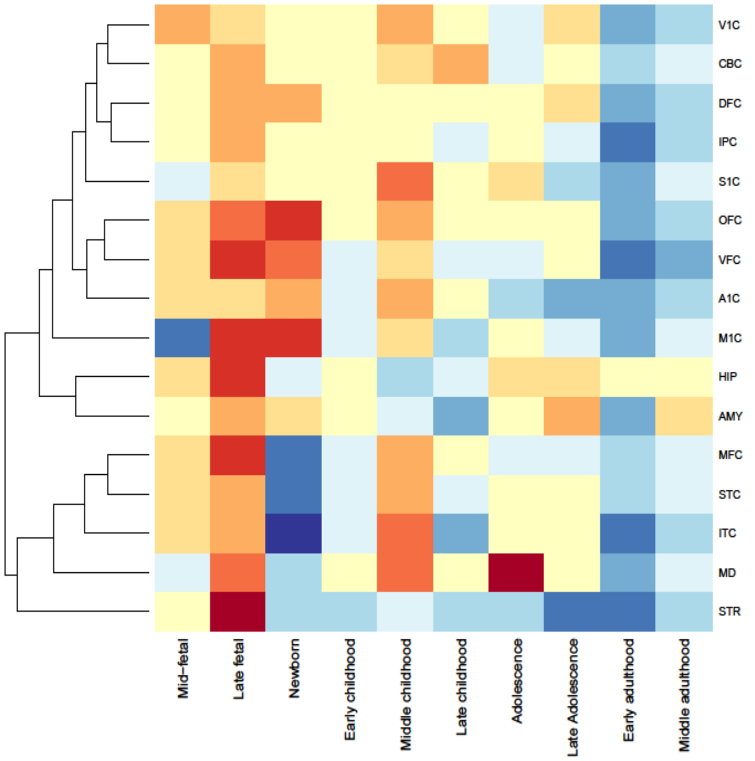

g) BIP & T2D

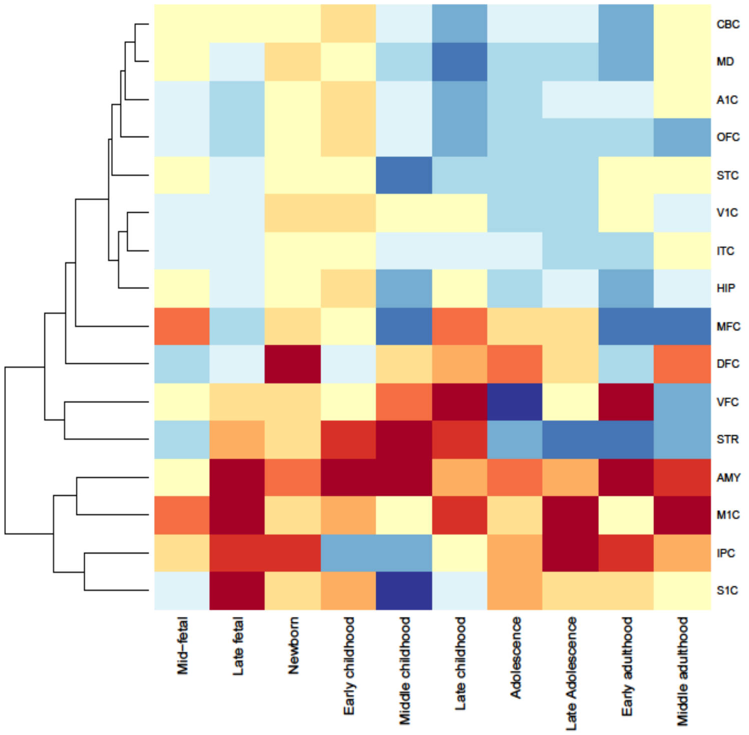

h) BIP & CAD

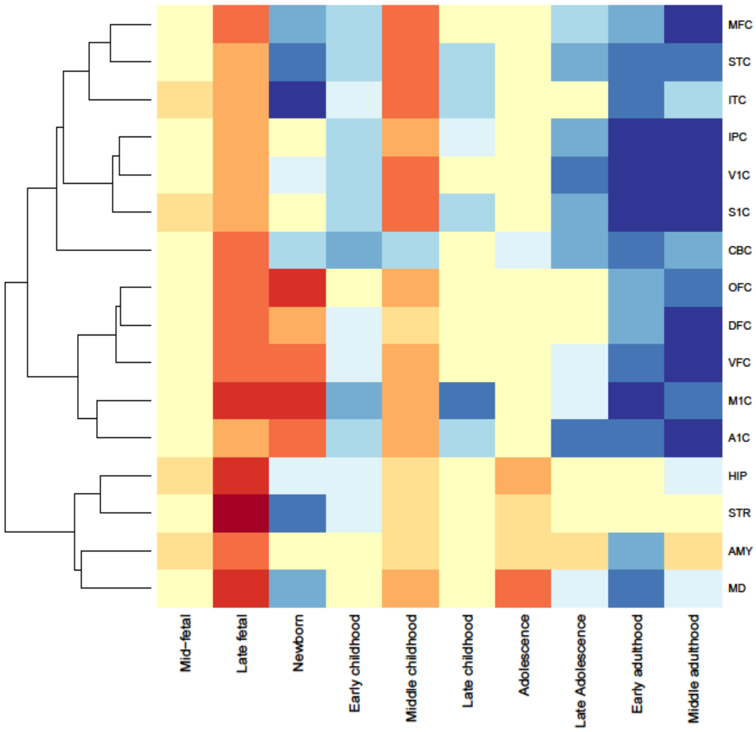

Supplementary Figure 8. Spatio-temporal gene expression for all genes mapped to candidate SNPs associated with bipolar disorder (BIP) and each CVD phenotype, including **a)** body mass index (BMI), **b)** systolic blood pressure (SBP), **c)** diastolic blood pressure (DBP), **d)** total cholesterol (TC), **e)** low-density lipoprotein (LDL) cholesterol, **f)** high-density lipoprotein (HDL) cholesterol, **g)** type 2 diabetes (T2D), **h)** coronary artery disease (CAD). Period of development on the x axis and neuroanatomical region on the y axis. We used RNA-Seq data from Brainspan and Weighted gene co-expression network analysis. Brain regions abbreviations: S1C: Primary Somatosensory Cortex; STC: Superior Temporal Cortex; ITC: Inferior Temporal Cortex; MFC: Medial Prefrontal Cortex; OFC: Orbital Prefrontal Cortex; M1C: Primary Motor Cortex; V1C: Primary Visual Cortex; VFC: Ventrolateral Prefrontal Cortex; IPC: Posterior Inferior Parietal Cortex; A1C: Primary Auditory Cortex; DFC: Dorsolateral Prefrontal Cortex; AMY: Amygdala; HIP: Hippocampus; CBC: Cerebellar Cortex; MD: Mediodorsal Nucleus of the Thalamus; STR: Striatum.

References

1. Stahl EA *et al.* Genome-wide association study identifies 30 loci associated with bipolar disorder. *Nature genetics* 2019; **51**(5)**:** 793-803.

2. Turcot V *et al.* Protein-altering variants associated with body mass index implicate pathways that control energy intake and expenditure in obesity. *Nat Genet* 2018; **50**(1)**:** 26-41.

3. Scott RA *et al.* An Expanded Genome-Wide Association Study of Type 2 Diabetes in Europeans. *Diabetes* 2017; **66**(11)**:** 2888-2902.

4. Willer CJ *et al.* Discovery and refinement of loci associated with lipid levels. *Nat Genet* 2013; **45**(11)**:** 1274-1283.

5. Evangelou E *et al.* Genetic analysis of over 1 million people identifies 535 new loci associated with blood pressure traits. *Nature genetics* 2018; **50**(10)**:** 1412-1425.

6. Nelson CP *et al.* Association analyses based on false discovery rate implicate new loci for coronary artery disease. *Nat Genet* 2017; **49**(9)**:** 1385-1391.

7. Bahrami S *et al.* Shared Genetic Loci Between Body Mass Index and Major Psychiatric Disorders: A Genome-wide Association Study. *JAMA Psychiatry* 2020.

8. Frei O *et al.* Bivariate causal mixture model quantifies polygenic overlap between complex traits beyond genetic correlation. *Nature communications* 2019; **10**(1)**:** 2417-2417.

9. Holland D *et al.* Beyond SNP heritability: Polygenicity and discoverability of phenotypes estimated with a univariate Gaussian mixture model. *PLOS Genetics* 2020; **16**(5)**:** e1008612.

10. Bulik-Sullivan BK *et al.* LD Score regression distinguishes confounding from polygenicity in genome-wide association studies. *Nature Genetics* 2015; **47**(3)**:** 291-295.

11. Benjamini Y, Hochberg Y. Controlling the False Discovery Rate: A Practical and Powerful Approach to Multiple Testing. *Journal of the Royal Statistical Society. Series B (Methodological)*, vol. 57. Blackwell Publishing1995, pp 289-300.

12. Efron B. Size, power and false discovery rates. *The Annals of Statistics* 2007; **35**(4)**:** 1351–1377.

13. Purcell S *et al.* PLINK: a tool set for whole-genome association and population-based linkage analyses. *American journal of human genetics* 2007; **81**(3)**:** 559-575.

14. Schweder T, Spjotvoll E. Plots of P-Values to Evaluate Many Tests Simultaneously. *Biometrika* 1982; **69**(3)**:** 493-502.

15. Andreassen OA *et al.* Improved detection of common variants associated with schizophrenia by leveraging pleiotropy with cardiovascular-disease risk factors. *American journal of human genetics* 2013; **92**(2)**:** 197-209.

16. Andreassen OA *et al.* Improved detection of common variants associated with schizophrenia and bipolar disorder using pleiotropy-informed conditional false discovery rate. *PLoS genetics* 2013; **9**(4)**:** e1003455.

17. Andreassen OA *et al.* Genetic pleiotropy between multiple sclerosis and schizophrenia but not bipolar disorder: differential involvement of immune-related gene loci. *Molecular psychiatry* 2015; **20**(2)**:** 207-214.

18. Andreassen OA, Thompson WK, Dale AM. Boosting the power of schizophrenia genetics by leveraging new statistical tools. *Schizophrenia bulletin* 2014; **40**(1)**:** 13-17.

19. Andreassen OA *et al.* Abundant genetic overlap between blood lipids and immune-mediated diseases indicates shared molecular genetic mechanisms. *PloS one* 2015; **10**(4)**:** e0123057.

20. Nichols T, Brett M, Andersson J, Wager T, Poline JB. Valid conjunction inference with the minimum statistic. *Neuroimage* 2005; **25**(3)**:** 653-660.

21. Schwartzman A, Lin X. The effect of correlation in false discovery rate estimation. *Biometrika* 2011; **98**(1)**:** 199-214.

22. Watanabe K, Taskesen E, van Bochoven A, Posthuma D. Functional mapping and annotation of genetic associations with FUMA. *Nat Commun* 2017; **8**(1)**:** 1826.

23. The 1000 Genomes Project Consortium. A global reference for human genetic variation. *Nature* 2015; **526**(7571)**:** 68-74.

24. Locke AE *et al.* Genetic studies of body mass index yield new insights for obesity biology. *Nature* 2015; **518**(7538)**:** 197-206.

25. Bulik-Sullivan B *et al.* An atlas of genetic correlations across human diseases and traits. *Nature Genetics* 2015; **47**(11)**:** 1236-1241.

26. Mullins N *et al.* Genome-wide association study of more than 40,000 bipolar disorder cases provides new insights into the underlying biology. *Nature Genetics* 2021; **53**(6)**:** 817-829.

27. MacArthur J *et al.* The new NHGRI-EBI Catalog of published genome-wide association studies (GWAS Catalog). *Nucleic Acids Res* 2017; **45**(D1)**:** D896-D901.

28. Smeland OB *et al.* Genome-wide analysis reveals extensive genetic overlap between schizophrenia, bipolar disorder, and intelligence. *Mol Psychiatry* 2019.

29. Drange OK *et al.* Genetic Overlap Between Alzheimer's Disease and Bipolar Disorder Implicates the MARK2 and VAC14 Genes. *Front Neurosci* 2019; **13:** 220.

30. O'Connell KS *et al.* Identification of genetic overlap and novel risk loci for attention-deficit/hyperactivity disorder and bipolar disorder. *Mol Psychiatry* 2019.

31. Andreassen OA *et al.* Improved detection of common variants associated with schizophrenia and bipolar disorder using pleiotropy-informed conditional false discovery rate. *PLoS Genet* 2013; **9**(4)**:** e1003455-e1003455.

32. Lee PH *et al.* Genomic Relationships, Novel Loci, and Pleiotropic Mechanisms across Eight Psychiatric Disorders. *Cell* 2019; **179**(7)**:** 1469-1482.e1411.

33. Genomic Dissection of Bipolar Disorder and Schizophrenia, Including 28 Subphenotypes. *Cell* 2018; **173**(7)**:** 1705-1715.e1716.

34. Hou L *et al.* Genome-wide association study of 40,000 individuals identifies two novel loci associated with bipolar disorder. *Hum Mol Genet* 2016; **25**(15)**:** 3383-3394.

35. Green EK *et al.* Replication of bipolar disorder susceptibility alleles and identification of two novel genome-wide significant associations in a new bipolar disorder case-control sample. *Mol Psychiatry* 2013; **18**(12)**:** 1302-1307.

36. Ikeda M *et al.* A genome-wide association study identifies two novel susceptibility loci and trans population polygenicity associated with bipolar disorder. *Mol Psychiatry* 2018; **23**(3)**:** 639-647.

37. Qi X *et al.* An integrative analysis of genome-wide association study and regulatory SNP annotation datasets identified candidate genes for bipolar disorder. *Int J Bipolar Disord* 2020; **8**(1)**:** 6.

38. Chen DT *et al.* Genome-wide association study meta-analysis of European and Asian-ancestry samples identifies three novel loci associated with bipolar disorder. *Mol Psychiatry* 2013; **18**(2)**:** 195-205.

39. Ferreira MA *et al.* Collaborative genome-wide association analysis supports a role for ANK3 and CACNA1C in bipolar disorder. *Nature genetics* 2008; **40**(9)**:** 1056-1058.

40. Psychiatric GCBDWG. Large-scale genome-wide association analysis of bipolar disorder identifies a new susceptibility locus near ODZ4. *Nat Genet* 2011; **43**(10)**:** 977-983.

41. Gordovez FJA, McMahon FJ. The genetics of bipolar disorder. *Mol Psychiatry* 2020; **25**(3)**:** 544-559.

42. Rødevand L *et al.* Polygenic overlap and shared genetic loci between loneliness, severe mental disorders, and cardiovascular disease risk factors suggest shared molecular mechanisms. *Translational Psychiatry* 2021; **11**(1)**:** 3.

43. Wu Y *et al.* Multi-trait analysis for genome-wide association study of five psychiatric disorders. *Transl Psychiatry* 2020; **10**(1)**:** 209.

44. Li HJ *et al.* Novel Risk Loci Associated With Genetic Risk for Bipolar Disorder Among Han Chinese Individuals: A Genome-Wide Association Study and Meta-analysis. *JAMA Psychiatry* 2021; **78**(3)**:** 320-330.

45. Muntané G *et al.* The shared genetic architecture of schizophrenia, bipolar disorder and lifespan. *Human Genetics* 2021; **140**(3)**:** 441-455.

46. Kircher M *et al.* A general framework for estimating the relative pathogenicity of human genetic variants. *Nat Genet* 2014; **46**(3)**:** 310-315.

47. Boyle AP *et al.* Annotation of functional variation in personal genomes using RegulomeDB. *Genome Res* 2012; **22**(9)**:** 1790-1797.

48. Roadmap Epigenomics C *et al.* Integrative analysis of 111 reference human epigenomes. *Nature* 2015; **518**(7539)**:** 317-330.

49. Zhu Z *et al.* Integration of summary data from GWAS and eQTL studies predicts complex trait gene targets. *Nat Genet* 2016; **48**(5)**:** 481-487.

50. Schmitt AD *et al.* A Compendium of Chromatin Contact Maps Reveals Spatially Active Regions in the Human Genome. *Cell Rep* 2016; **17**(8)**:** 2042-2059.

51. Ashburner M *et al.* Gene ontology: tool for the unification of biology. The Gene Ontology Consortium. *Nat Genet* 2000; **25**(1)**:** 25-29.

52. Kamburov A, Stelzl U, Lehrach H, Herwig R. The ConsensusPathDB interaction database: 2013 update. *Nucleic Acids Research* 2012; **41**(D1)**:** D793-D800.

53. Bahrami S *et al.* Genetic loci shared between major depression and intelligence with mixed directions of effect. *Nature Human Behaviour* 2021.

54. Johnson MB *et al.* Functional and evolutionary insights into human brain development through global transcriptome analysis. *Neuron* 2009; **62**(4)**:** 494-509.

55. Colantuoni C *et al.* Temporal dynamics and genetic control of transcription in the human prefrontal cortex. *Nature* 2011; **478**(7370)**:** 519-523.

56. Kang HJ *et al.* Spatio-temporal transcriptome of the human brain. *Nature* 2011; **478**(7370)**:** 483-489.

57. Langfelder P, Horvath S. WGCNA: an R package for weighted correlation network analysis. *BMC Bioinformatics* 2008; **9:** 559.
